# Comparative gene expression profile analysis of ovules provides insights into *Jatropha curcas* L. ovule development

**DOI:** 10.1038/s41598-019-52421-0

**Published:** 2019-11-04

**Authors:** Gang Xu, Jian Huang, Shi-kang Lei, Xue-guang Sun, Xue Li

**Affiliations:** 1 Institute for Forest Resources and Environment of Guizhou / College of Forestry, Guizhou University, Guiyang, 550025 P.R. China; 2 School of Life Science, Guizhou University, Guiyang, Guizhou P.R. China; 3 Key Laboratory of Green Pesticide and Agricultural Bioengineering, Ministry of Education, Center for Research and Development of Fine Chemicals of Guizhou University, Guiyang, Guizhou P.R. China; 4 Institute of Entomology, Guizhou University, Guiyang, Guizhou P.R. China

**Keywords:** Plant regeneration, Plant physiology

## Abstract

*Jatropha curcas*, an economically important biofuel feedstock with oil-rich seeds, has attracted considerable attention among researchers in recent years. Nevertheless, valuable information on the yield component of this plant, particularly regarding ovule development, remains scarce. In this study, transcriptome profiles of anther and ovule development were established to investigate the ovule development mechanism of *J*. *curcas*. In total, 64,325 unigenes with annotation were obtained, and 1723 differentially expressed genes (DEGs) were identified between different stages. The DEG analysis showed the participation of five transcription factor families (bHLH, WRKY, MYB, *NAC* and ERF), five hormone signaling pathways (auxin, gibberellic acid (GA), cytokinin, brassinosteroids (BR) and jasmonic acid (JA)), five MADS-box genes (*AGAMOUS*-2, *AGAMOUS*-1, *AGL1*, *AGL11*, and *AGL14*), *SUP* and *SLK3* in ovule development. The role of GA and JA in ovule development was evident with increases in flower buds during ovule development: GA was increased approximately twofold, and JA was increased approximately sevenfold. In addition, the expression pattern analysis using qRT-PCR revealed that *CRABS CLAW* and *AGAMOUS*-2 were also involved in ovule development. The upregulation of BR signaling genes during ovule development might have been regulated by other phytohormone signaling pathways through crosstalk. This study provides a valuable framework for investigating the regulatory networks of ovule development in *J*. *curcas*.

## Introduction

*Jatropha curcas* L., a species native to tropical regions in the Western Hemisphere, is now found prevalently distributed in Africa and Asia. *J*. *curcas* is described as an ideal bioenergy crop for its oil-rich seeds, high unsaturated fatty acid content in seed oil (the oil with high unsaturated fatty acid content is suitable for producing biodiesel), low nutrient requirements and high drought tolerance. In addition, *J*. *curcas* also has potential in medical applications such as anti-tumor, anti-microbial and anti-parasitic^1–3^. However, due to low seed yield, this plant has limited economic benefit for exploitation and further expansion of the Jatropha-based biodiesel industry. The low ratio of female to male flowers (1/10—1/30) is one of the critical factors attributed to the low seed yield of *J*. *curcas*^4^; therefore, in *J*. *curcas*, gene expression profile analysis was employed to study the development of seeds^5^, the response of seedlings to drought and salt stress^6,7^, and lipid metabolism in seeds and other tissues^8^. For high-throughput discovery of novel Jatropha genes, *de novo* assembly and transcriptome analysis of various tissues of *J*. *curcas* were performed, obtaining 17,457 assembled transcripts (contigs) and 54,002 singletons^9^. However, genomics studies on flower development in *J*. *curcas* are relatively scarce. Pan *et al*. analyzed the transcriptome of the inflorescence meristems of *J curcas* treated with cytokinin, obtained 81,736 unigenes, and identified a series of cytokinin-responsive genes, such as *JcCycA3;2*, *JcCycD3;1*, *JcCycD3;2* and *JcTSO1*, which were thought to contribute to the increase in flower number^10^. Xu *et al*. analyzed the transcriptome of flower buds at different phases of sex differentiation in *J*. *curcas*, obtained 57962 unigenes, and found that the gibberellin-regulated protein 4-like gene and the AMP-activated protein kinase gene are associated with stamen differentiation^11^.

*J*. *curcas* is a monoecious plant. Sex differentiation occurs after the initiation of five petal primordia. Unlike male flowers, the female flowers undergo a hermaphrodite period: stamens first develop carpels and then gradually degenerate as the three carpels to be formed are fused^11^. Ovule development is an important process for the development of female flowers and is crucial for fruit yield. Any disturbances during macrosporogenesis or female gametogenesis may result in female flower abortion. Molecular studies on sporogenesis and gametogenesis of *J*. *curcas* are not available; therefore, transcriptome profiles spanning the developmental processes of pollen and ovule in *J*. *curcas* were constructed. In this study, we focused on macrosporogenesis and female gametogenesis during ovule development, and differentially expressed gene analysis was then performed on the expression profiles of flower buds at different developmental stages of the ovule in female flowers.

## Results

### Development of the ovule

**Figure 1:**
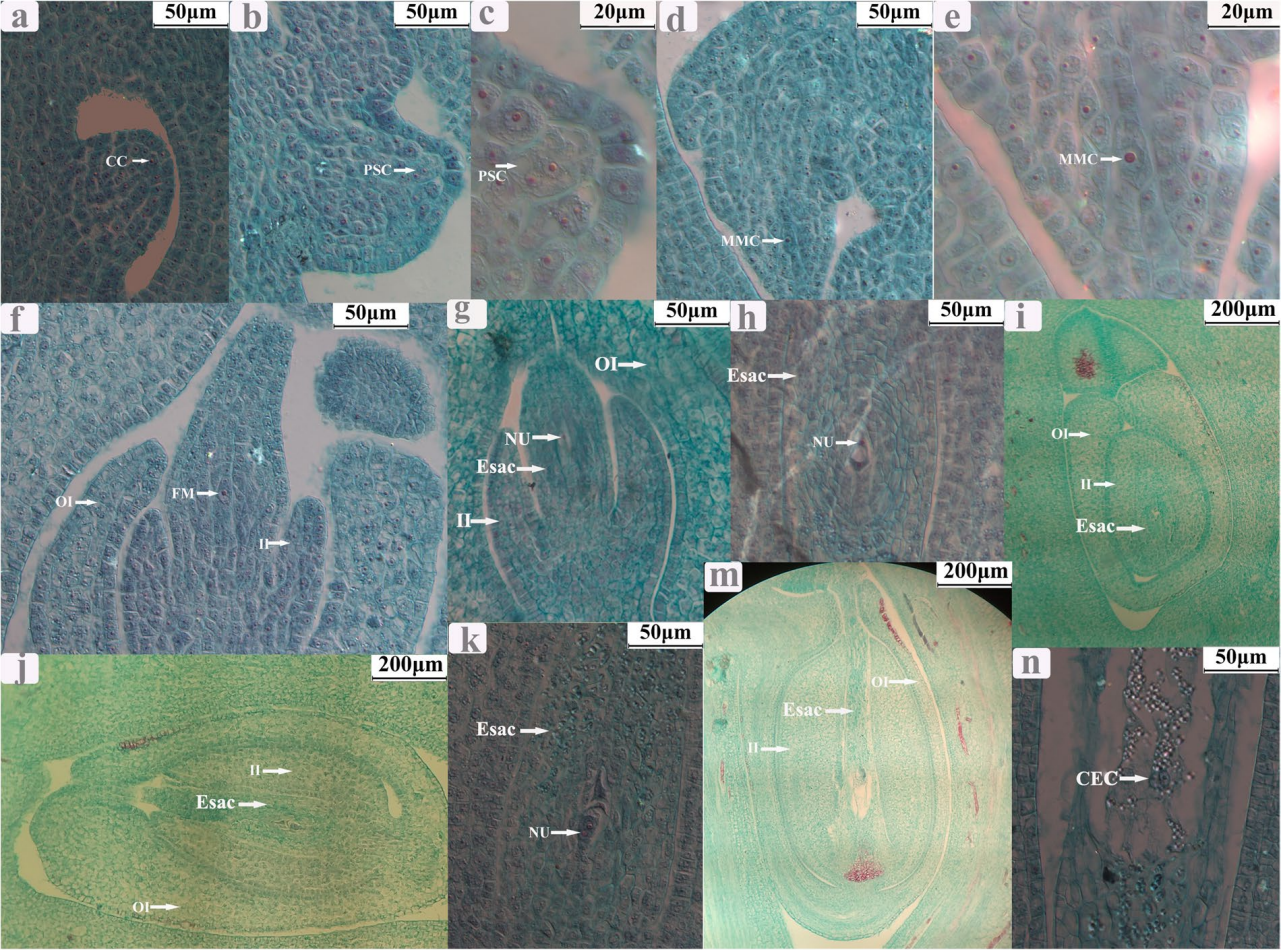
Microstructure of ovule at different development stages in *Jatropha curcas* female flowers. (**a**) Chesporial cell; (**b**,**c**) the occurance of primary sporogenous cells and a parietal cell; (**d**,**e**) the occurance of macrospore mother cell; (**f**,**g**) the formation of functional macrospore (mononuclear embryo sac); (**h**,**i**) the formation of 2-nucleate embryo sac; (**j**,**k**) the formation of 8- nucleate embryo sac; (**m**,**n**) the maturation of embryo sac. CC: Chesporial cell; PSC: primary sporogenous cells; PC: parietal cell; MMC: macrospore mother cell; Esac: embryo sac; NU: nucleate; II: inner integuments; OI: outer integuments; CEC: Central cell.

**Figure 2:**
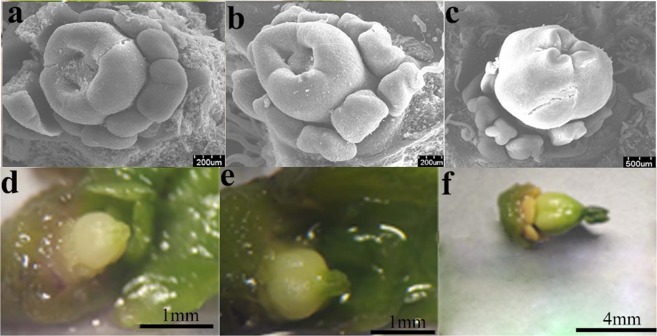
The morphology of ovule (or cuples) at different development stages. JCFI: from (**a** to **b**); JCFII: from (**c** to **d**); JCFIII: from (**d** to **f**); JCFIV: from (**f** to **e)**.

After the fusion of the three carpels, the ovule primordium was formed. When the ovary was approximately 0.3 mm long, archesporial cells occurred. When the ovary was approximately 0.6 mm long, archesporial cells gave rise to primary sporogenous cells. When the ovary was approximately 0.9 mm long, the style began to develop, and the primary sporogenous cells developed into macrospore mother cells (MMC). When the ovary was approximately 1.2 mm long, the style was deep green, and the stigmas appeared; MCC underwent two consecutive meioses with a result of 4 cells, of which three were degenerated, and the remaining one was developed into a functional macrospore. When the ovary was approximately 3.2 mm long, functional macrospores experienced three consecutive mitoses, resulting in an embryo sac with 8 nuclei. When the whole ovary became green, the stigmas were mature; of these 8 nuclei, two moved to the center of the embryo sac and formed a polar, and the remaining 6 formed an egg, two synergids, and three antipodal cells, respectively (Figs 1 and 2).

### Endogenous phytohormone level in flower buds at different development stages

**Figure 3:**
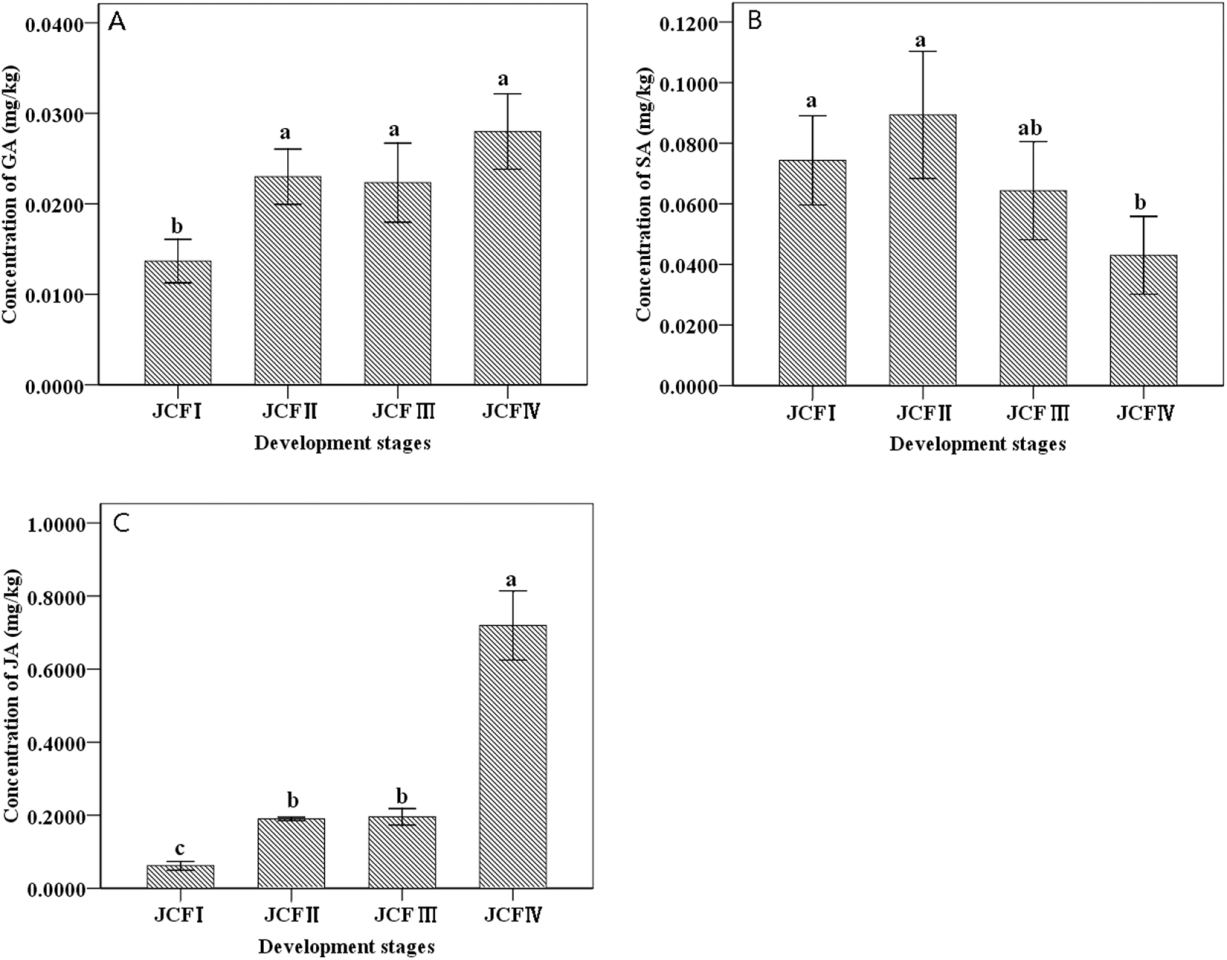
The concentration of endogenous gibberellic acid (GA), jasmonic acid (JA) and salicylic acid (SA) in flower buds from different stages of ovule development.

The concentrations of endogenous gibberellic acid (GA) and jasmonic acid (JA) in flower buds began to significantly increase in JCFII, but JA further experienced a dramatic increase in JCFIV. Additionally, the concentration of endogenous SA in flower buds significantly declined only at the stage of JCFIV and only slightly changed in the stages of JCFl, JCFII and JCFIII (Fig. 3).

### Identification of differently expressed genes

**Figure 4:**
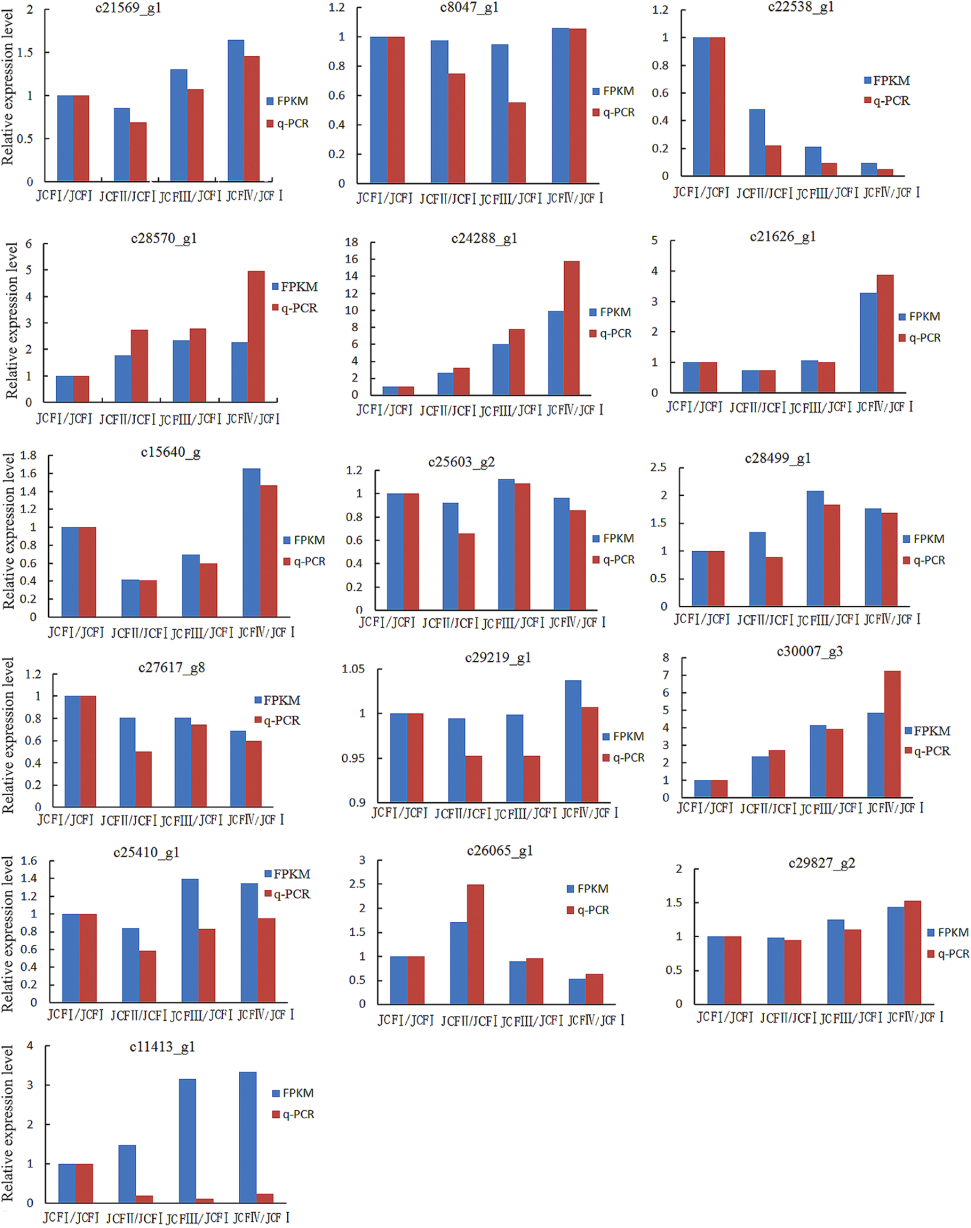
Genes differently expressed during ovule development.

**Figure 5:**
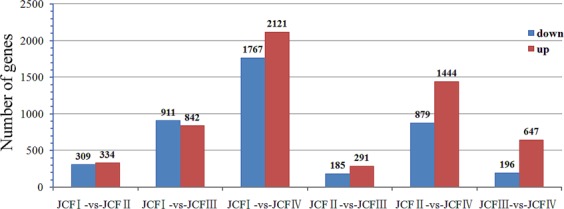
Venn diagram showing the overlaps between different develop stages of ovule.

The sequencing results of the twelve sample DGE libraries are shown in Table 1. The depth, coverage and homogenization of sequencing were high, which suggested that these results could reflect the actual expression of genes, and the data were thus suitable for further analysis (Fig. S2). In these twelve libraries, approximately 83.78–87.00% of the clean reads could be mapped to the assembled transcriptome (Table S4). In total, 1723 genes that were differentially expressed during ovule development in female flower buds were screened out and are shown in Fig. 4. Of these differentially expressed genes (DEGs), 9 genes were downregulated and 12 genes were upregulated throughout the entire process of ovule development; 579 genes were downregulated and 1036 genes were upregulated in one stage; 42 genes were downregulated and 100 genes were upregulated at two stages (Fig. 5).

**Table 1:** Expression level of ovule development related genes during ovule development.

Gene_id	Annotation description	Protein ID	log2(JCFII/JCFI)	log2(JCFIII/JCFII)	log2(JCFIV/JCFIII)	log2(JCFIII/JCFI)	log2(JCFIV/JCFI)	log2(JCFIV/JCFII)
c26856_g5	*Argonaute 2* (*Arabidopsis thaliana*)	Q9SHF3					0.692	0.814
c25989_g2	*AGAMOUS* (*AGAMOUS-1*) (*Panax ginseng*)	Q40872	0.902			1.407	1.178	
c27618_g1	*SLK3* (*Arabidopsis thaliana*)	F4JT98			0.618	0.561	1.179	1.023
c29108_g3	*AGL14*(*Arabidopsis thaliana*)	Q38838	1.073			1.895	1.742	0.670
c30128_g4	*AGL11*(*Arabidopsis thaliana*)	Q38836	2.751	1.351		4.105	4.115	1.367
c25989_g3	*AGL1*(*Arabidopsis thaliana*)	P29381	1.126			1.470	1.544	
c24381_g1	*YABBY 5* (*Arabidopsis thaliana*)	Q8GW46		0.660	0.650	0.809	1.459	1.316
c21190_g1	*SUPERMAN* (*Arabidopsis thaliana*)	Q38895	1.477			1.329		
c24288_g1	*AGAMOUS* (*AGAMOUS-2*) (*Nicotiana tabacum*)	Q43585	1.329	1.210	0.730	2.541	3.271	1.946

### Gene expression patterns of selected genes

**Figure 6:**
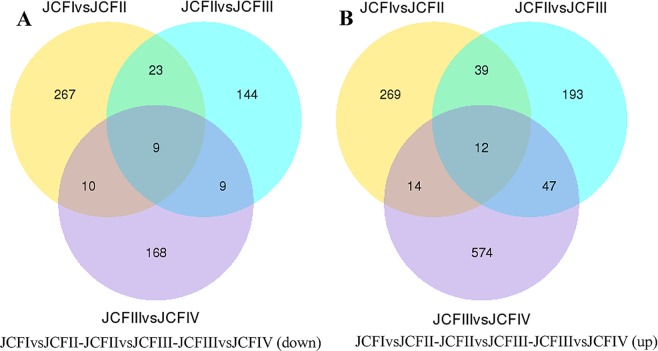
Expression pattern of randomly selected genes. The fold changes of the genes were calculated as the ratio of the JCFI/JCFI, JCFII/JCFI, JCFIII/JCFI and JCFIV/JCFI and are shown on the y-axis.

In total, 16 DEGs were selected for qRT-PCR analysis to validate the expression profiles obtained by digital gene expression analysis, and the results showed that the expression patterns of these genes were consistent with the results obtained by digital gene expression analysis, except AUX22 (c11413_g1) (Fig. 6), indicating that transcriptome data in this study were reliable.

### KEGG pathway enrichment analysis of differentially expressed genes

**Figure 7:**
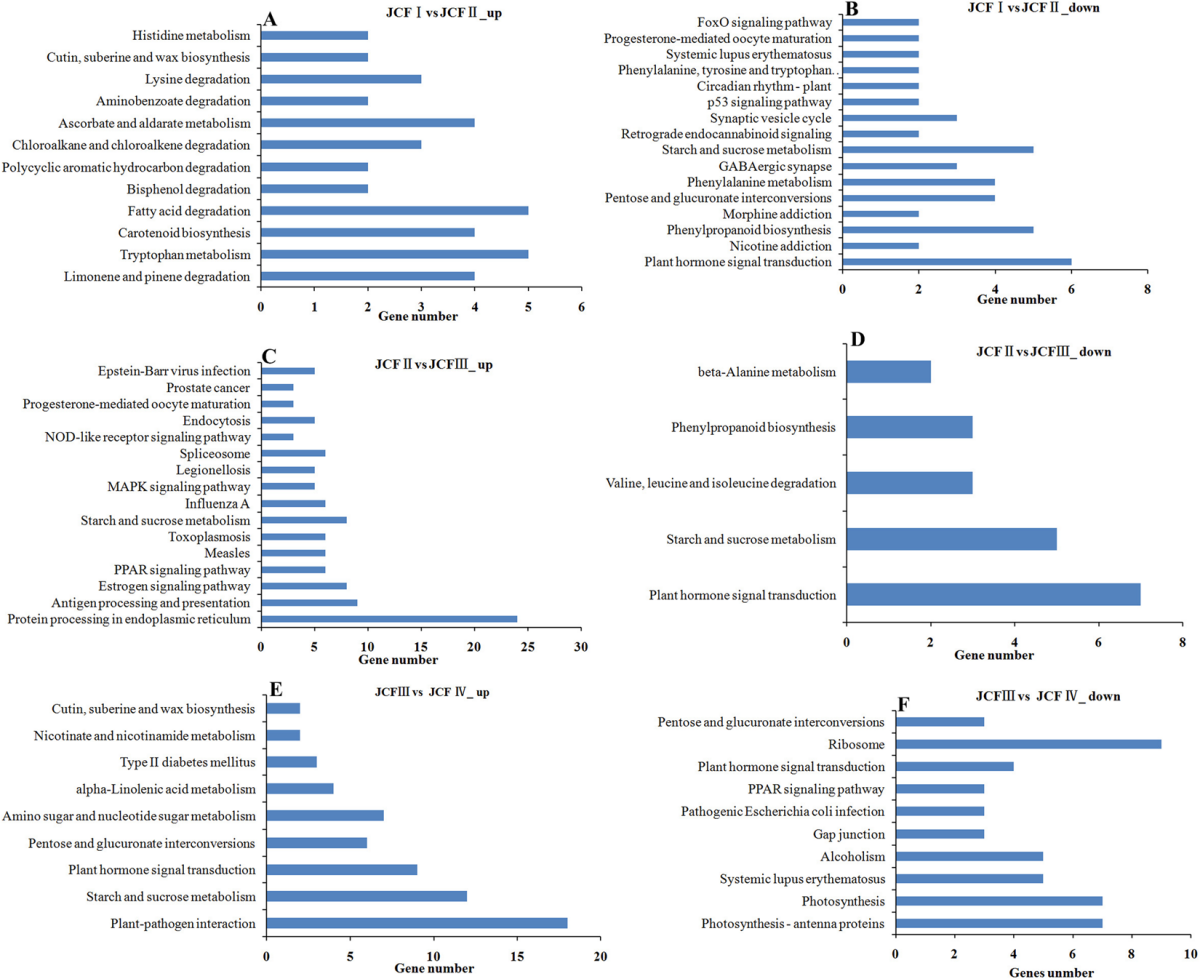
KEGG classification analysis of the differently expressed genes.

After KEGG analysis, the significantly enriched pathways were selected for further analysis. The results indicated that sugar metabolism and protein biosynthesis/processing were respectively enhanced during the maturation of ES and the formation of MES, and the metabolisms of auxin and JA were also upregulated during the formation of MMC and the maturation of EC, but the metabolism of SA declined during ovule development. Among the significantly enriched pathways (Fig. 7), plant hormone signal transduction was downregulated in pairwise JCFI vs JCFII and JCFII vs JCFIII but showed a trend to upregulate in pairwise JCFIII vs JCFIV; starch and sucrose metabolism was upregulated in pairwise JCFIII vs JCFIV; protein processing in endoplasmic reticulum was upregulated in pairwise JCFIII vs JCFII; tryptophan metabolism associated with auxin metabolism was upregulated in pairwise JCFI vs JCFII; alpha-linolenic acid metabolism associated with JA metabolism was upregulated in pairwise JCFIII vs JCFIV; phenylalanine metabolism associated with SA metabolism was downregulated in pairwise JCFI vs JCFII.

### Differentially expressed transcription factor genes during ovule development

**Figure 8:**
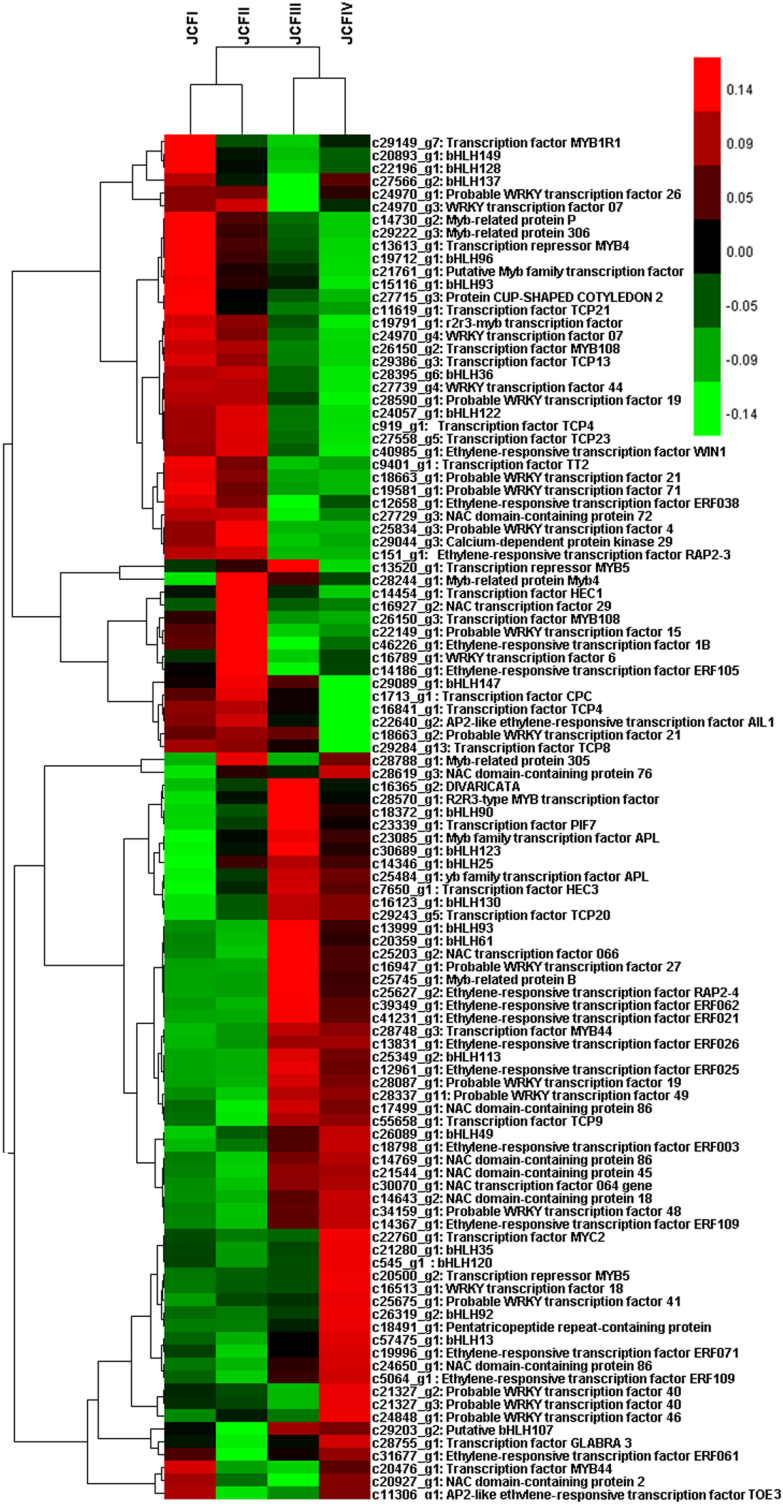
Cluster analysis showing the differentially expressed transcriptor factor genes during ovule development.

**Figure 9:**
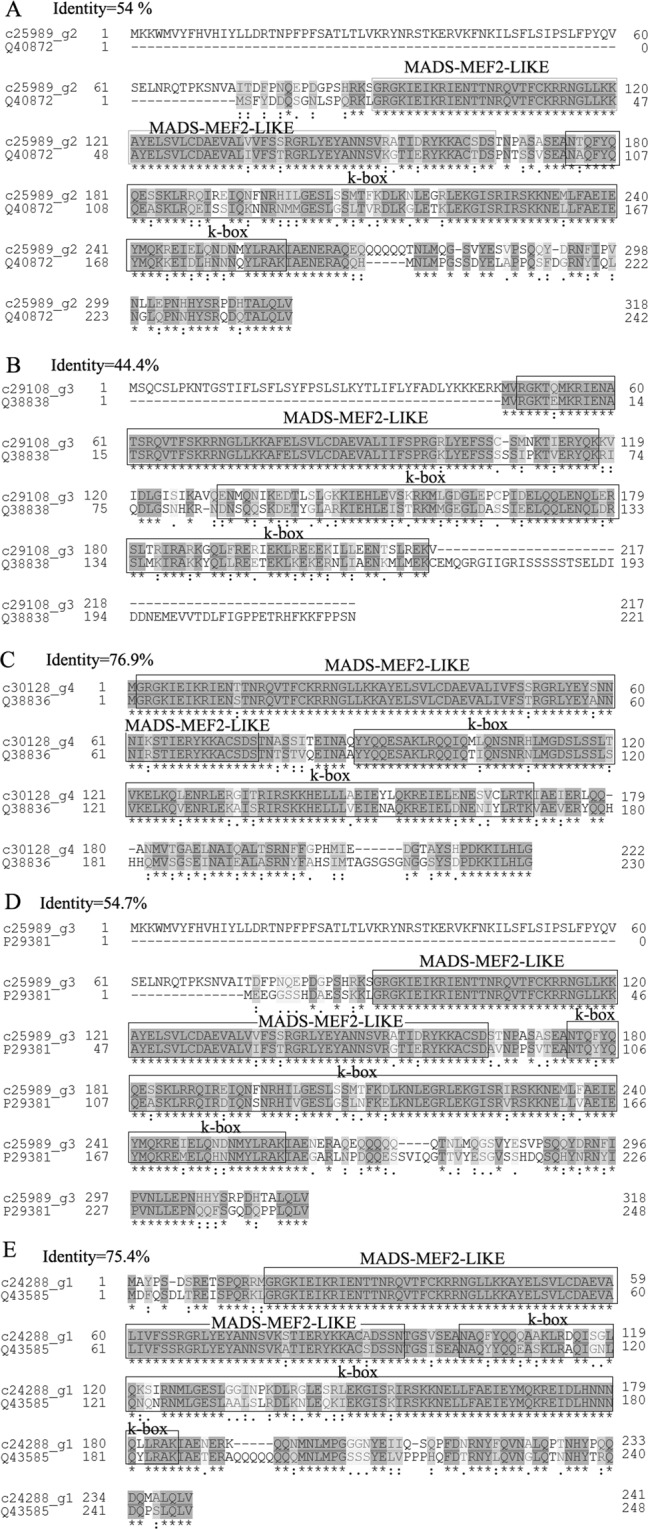
Sequence alignment between five MADS-box proteins and their homologues in protein database UniProt.

A total of 156 transcription factor (TF) genes were differentially expressed during ovule development, of which the top six TF families were *bHLH*, *WRKY*, *MYB*, ethylene-responsive transcription factors (*ERF*), *NAC* and *TCP*: 26 from *bHLH*, 21 from *WRKY*, 20 from *MYB*, 18 from *ERF*, 13 from *NAC*, and 8 from *TCP*. Among the DEGs of these six TF families, the upregulated TF genes included 17 *bHLH*, 11 *WRKY*, 12 ERF, 10 *NAC*, 10 *MYB* and 2 *TCP* (Fig. 8). Of these 10 *MYB* genes, a *R2R3*-type *MYB* gene (c28570_g1) was confirmed to be upregulated with the development of the ovule by qRT-PCR analysis (Fig. 9). These results suggested that *bHLHs*, *WRKYs*, ERFs, *NACs* and *MYBs* would extensively participate in ovule development.

### Prediction of gene regulating ovule development

**Figure 10:**
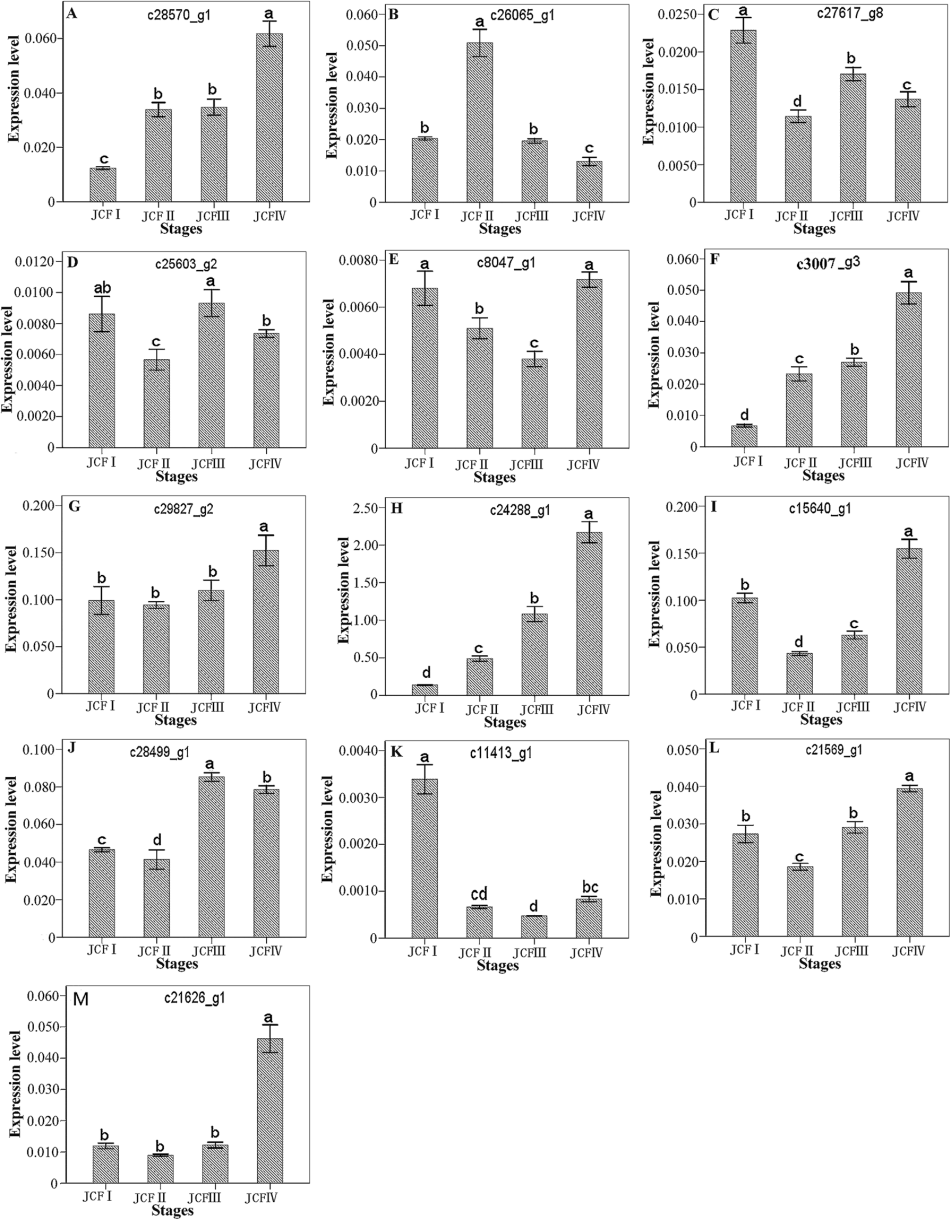
One-way ANOVA analysis of expression level of 15 selected genes in samples at different ovule development stages, as detected by real-time QPCR. Different letters in the same row indicate significant differences (P ≤ 0.005). c28570_g1: R2R3-type MYB; c30007_g3: CRABS CLAW; c24288_g1: AGAMOUS-2; C28499_g1: Auxin-induced protein 5NG4; c21569_g1: IAA1; c15640_g1: GASA3; c8047_g1: BZR1; C21626_g1: Auxin-induced in root cultures protein 12; c29827_g2: GAI; c26065_g1: GASL7; c27617_g8: ARF5; c25603_g2: BAK1; c11413_g1: AUX22.

In total, 9 genes annotated as ovule development-related genes were upregulated during ovule formation as suggested by transcriptome analysis, including 5 MADS-box protein genes (*AGAMOUS-2* (*AG-2*), *AG-1*, *AGL1*/*SHP1*, *AGL11*/*STK* and *AGL14*), *YABBY 5*, *UPERMAN (SUP*), *SLK3* and *Argonaute* 2 (Table 1). These five MADS-box proteins were then subjected to sequence alignment with their homologs in the protein database UniProt. The results showed that these five MADS-box proteins have a MADS-MEF2-LIKE and a K-box domain and belong to the MADS-MEF2-LIKE subfamily of MADS (Fig. 9). The MADS-MEF2-LIKE domain showed high homology between these five MADS-box proteins and their homologs (Fig. 9). Of these 9 genes, *AG-2* was upregulated throughout ovule development, as suggested by both transcriptome analysis and qRT-PCR analysis (Fig. 10; Table 1); *AGL1*, *AGL14*, *AG-1* and *SUP* were upregulated in pairwise JCFI vs JCFII; *SLK3* was upregulated in pairwise JCFIV vs JCFIII; *AGL11*/*STK* was continuously upregulated from JCFI to JCFIII; *YABBY 5* was continuously upregulated from JCFII to JCFIV; argonaute 2 was upregulated in pairwise JCFII vs JCFIV and JCFI vs JCFIV. On the other hand, *CRABS CLAW* (*CRC*), annotated as an ovule development-related gene, was upregulated from JCFI to JCFIV, as suggested by qRT-PCR analysis (Fig. 10).

### Phytohormone signaling responsive genes associated with ovule development

**Figure 11:**
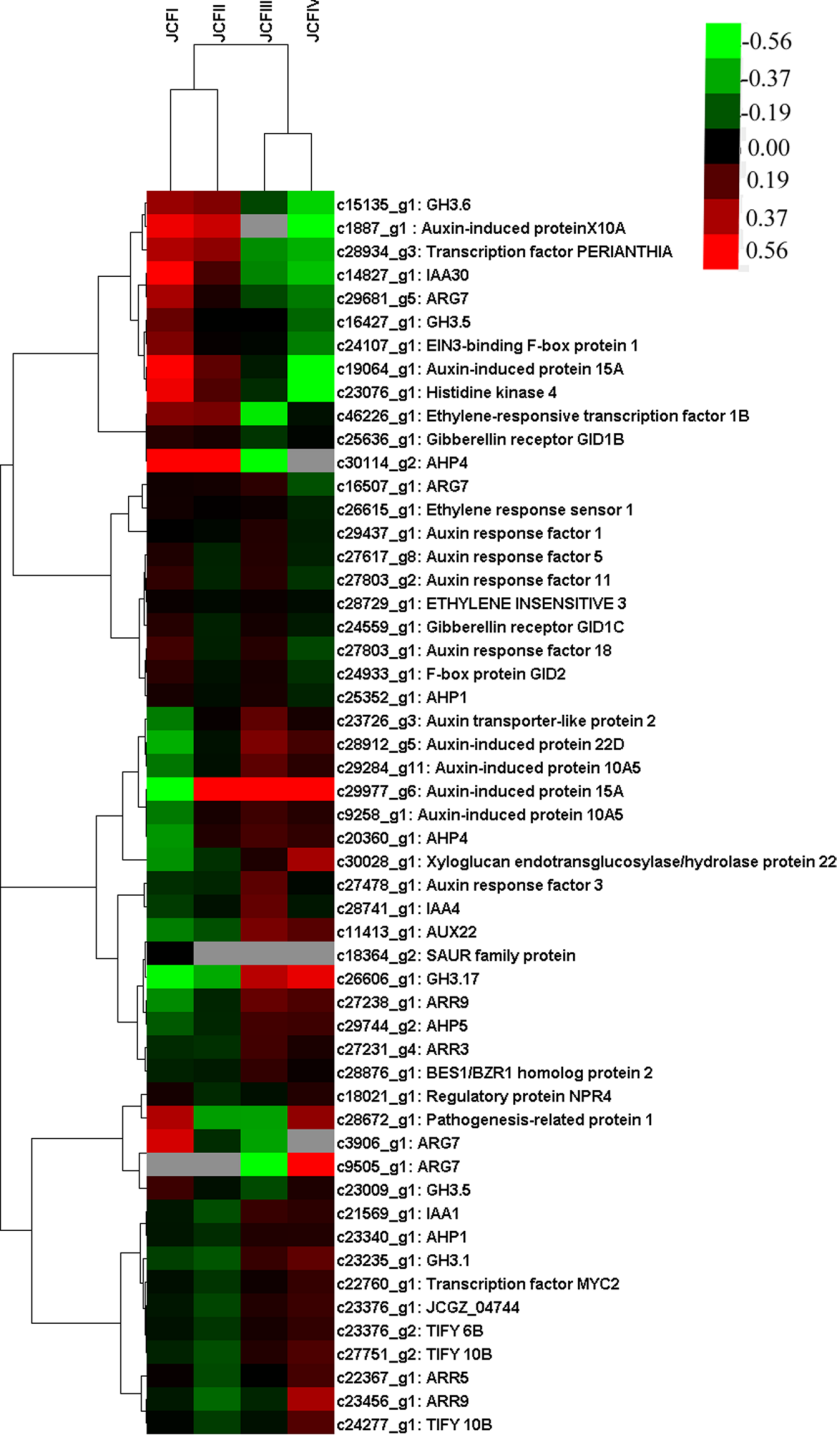
Cluster analysis showing the differentially expressed genes enriched in phytohormone signaling during ovule development.

In all, 71 phytohormone signaling-responsive DEGs were identified, 61 of which were screened out by transcriptome analysis, 9 by qRT-PCR analysis, and *IAA1* by both qRT-PCR and transcriptome analysis (Figs 10, 11). The expression of *IAA1* was first downregulated at JCFII and then significantly upregulated during the following development stages (Fig. 10B,L).

Of these 61 DEGs screened out by transcriptome analysis, 24 were from auxin, 2 from BR, 10 from CK, 8 from ABA, 5 from ETH, 3 from GA, 6 from JA, and 3 from SA. In pairwise CFI vs JCFII, the expression of 4 *SAUR* genes (*22D* protein, *15A-1* and two *10A5* (*10A5-1* and *10A5-2)*) in auxin and histidine-containing phosphotransfer proteins 4 (*HP4-2*) in CK were upregulated, whereas *ARF 11*, *ARF 18* and *GH3*.*5* in auxin were downregulated. In pairwise JCFII vs JCFIII, the expression of *GH3*.*17* in auxin was upregulated, whereas *IAA30* and *X10A* in auxin and *GH3*.*5* in JA were downregulated. In pairwise JCFIII vs JCFIV, the expression of *GH3*.*1* in auxin, *ARR9-2* and *ARR5* in CK, gibberellin receptor *GID1B* in GA, xyloglucan endotransglucosylase/hydrolase protein 22 (*XTH22*) in BR, pathogenesis-related protein 1 in SA, and two *TIFY* 10B and a *MYC*2 in JA were upregulated, whereas *ARG7-3* in auxin was downregulated. In pairwise JCFI vs JCFIII, JCFI vs JCFIV or JCFII vs JCFIV, the expression of auxin transporter-like protein 2 (*ATLP2*), *IAA4*, *ARF3* and *ARG7-1* in auxin, *HP5*, *HP1-2*, *ARR3* and *ARR9-1* in CK, *BES1*/*BZR1* homolog protein 2 in BR, and *TIFY 6B* and hypothetical protein JCGZ_04744 in JA were upregulated, whereas *ARG7-2*, *ARG7-4*, *15A-2*, *GH3*.*6*, *ARF 5* and *ARF 1* in auxin, *GID1C* and *GID2* in GA, *HP1* in CK, *NPR4* and *PERIANTHIA* in SA were downregulated. On the other hand, *HK 4* was continuously downregulated during ovule development, and *HP4-1* was downregulated from JCFI to JCFIII. The genes in ETH signaling were all downregulated during the development of the ovule. Of the 8 genes involved in ABA signaling, only ABA receptor *PYL9* was downregulated in pairwise JCFIII vs JCFIV, while the other genes gradually changed their expression during ovule development (Fig. 11).

Of these 9 DEGs screened by qRT-PCR analysis, 3 genes (*GASA3*, *GAI* and *GASL7*) were from the GA response signaling pathway, 2 (BRASSINOSTEROID INSENSITIVE 1-associated receptor kinase 1 (*BAK1*) and (BRASSINAZOLE-RESISTANT 1 protein (*BZR1*)) from BR, 4 (*ARF5*, *5NG4*, *AUX22* and auxin-induced in root cultures protein 12 (*AIT12*)) from auxin. Of these genes, *GASA3* was upregulated from JCFII to JCFIV, and *GASL7* from JCFI to JCFII; *BZR1*, *AIT12* and *GAI* were upregulated from JCFIII to JCFIV; *5NG4*, *ARF5* and *BAK1* were from JCFII to JCFIII; *AUX22* was greatly downregulated at the stages after JCFI (Fig. 10).

### Differentially expressed genes related to phytohormone metabolism

The DEGs related to phytohormone metabolism were screened out as follows: auxin metabolism-related DEGs were screened out from the DEGs enriched in the tryptophan metabolism pathway, CK metabolism-related DEGs from the DEGs enriched in the enriched zeatin biosynthesis pathway, gibberellin metabolism-related DEGs from the DEGs enriched in the diterpenoid biosynthesis pathway, BR metabolism-related DEGs from the DEGs enriched in metabolism pathway, JA metabolism-related DEGs from the DEGs enriched in the alpha-linolenic acid metabolism pathway, and SA biosynthesis-related DEGs from the DEGs enriched in phenylalanine biosynthesis (Table 2).

**Table 2:** Phytohormone biosynthesis genes differently expressed during the ovule development.

Genes	Swissprot Description	Protein ID	log2(JCFII/JCFI)	log2(JCFIII/JCFII)	log2(JCFIV/JCFIII)	log2(JCFIII/JCFI)	log2(JCFIV/JCFI)	log2(JCFIV/JCFI I)
**Auxin**								
c24045_g2	*Probable YUCCA8 (Arabidopsis thaliana)*	Q9SVU0	1.504					
c25692_g3	*Aldehyde dehydrogenase family 3 member H1(ALDH3 H1) (Arabidopsis thaliana)*	Q70DU8	1.096					
c14699_g1	*Aldehyde dehydrogenase family 2 member B4(ALDH2 B4-1)*, *(Arabidopsis thaliana)*	Q9SU63	0.737					
c20408_g1	*Aldehyde dehydrogenase family 2 member B4(ALDH2 B4-2)*, *(Arabidopsis thaliana)*	Q9SU63		−0.688				
c29730_g3	*Cytosolic sulfotransferase 16 (Arabidopsis thaliana)*	Q9C9D0					−1.383	−0.884
c24704_g1	*Aldehyde dehydrogenase family 3 member F1(ALDH3 F1) (Arabidopsis thaliana)*	Q70E96					−0.625	
c26913_g1	*YUCCA2 (Arabidopsis thaliana)*	Q9SVQ1						−2.025
c28407_g1	*YUCCA4 (Arabidopsis thaliana)*	Q9LFM5						−0.937
**Gibberellins**								
c15206_g1	*Gibberellin 2-beta-dioxygenase 2 (Pisum sativum)*	Q9XHM5		−2.105				−2.100
c25340_g1	*Ent-kaurene oxidase (Arabidopsis thaliana)*	Q93ZB2		0.749		1.394	2.109	1.469
c28071_g1	*Ent-kaurenoic acid oxidase 1 (Arabidopsis thaliana)*	O23051				0.558	0.585	
**Cytokinin**								
c39116_g1	*Cytokinin dehydrogenase 3 (Arabidopsis thaliana)*	Q9LTS3	−2.138					
c29016_g9	*Adenylate isopentenyltransferase 5 (Arabidopsis thaliana)*	Q94ID2	2.246		1.099	2.785	3.884	1.642
**JA**								
c27373_g1	*3-ketoacyl-CoA thiolase 2 (Arabidopsis thaliana)*	Q56WD9		0.746	1.155		1.679	1.905
c27860_g2	*Linoleate 13S-lipoxygenase 3-1 (Solanum tuberosum)*	O24371			1.015		1.275	1.182
c17968_g1	*Allene oxide synthase (Linum usitatissimum)*	P48417			1.385		1.404	1.483
c29952_g2	*12-oxophytodienoate reductase 3 (Solanum lycopersicum)*	Q9FEW9			1.040			0.957
c29272_g6	*Acyl-coenzyme A oxidase 2 (Arabidopsis thaliana)*	O65201					−0.733	
c29400_g1	*fatty acid beta-oxidation multifunctional protein AIM1 (Arabidopsis thaliana)*	Q9ZPI6				0.522	0.942	0.723
c24535_g1	*4-coumarate–CoA ligase-like 5 (Arabidopsis thaliana)*	Q84P21					0.520	0.476
c27608_g6	*Allene oxide cyclase 4 (Arabidopsis thaliana)*	Q93ZC5					1.095	1.203
c28745_g1	*Linoleate 13S-lipoxygenase 2-1 (Solanum tuberosum)*	O24370					1.350	0.903
c29303_g1	*Putative 12-oxophytodienoate reductase 11 (Oryza sativa subsp*. *Japonica)*	B9FSC8						
**SA**								
c29339_g2	*Phenylalanine ammonia-lyase (Populus trichocarpa)*	P45730					−1.040	−0.617
**BR**								
c28090_g2	*CYP90D1 (Arabidopsis thaliana)*	Q9M066						−0.672
c24187_g1	*CYP90B1 (Arabidopsis thaliana)*	O64989				−0.990		

In total, 26 differentially expressed genes were identified, of which 8 were from auxin, 2 from BR, 2 from CK, 3 from GA, 10 from JA, and 1 from SA. In pairwise JCFI vs JCFII, the expression levels of YUCCA8, aldehyde dehydrogenase family 3 member H1 (*ALDH 3H1*) and *ALDH 2B4-1* in auxin were upregulated, whereas cytokinin dehydrogenase 3 in CK was downregulated. In pairwise JCFII vs JCFIII, the expression of Ent-kaurene oxidase (KO) in GA was upregulated, whereas *ALDH 2 B4-2* in auxin and gibberellin 2-beta-dioxygenase 2 in GA were downregulated. In pairwise JCFIII vs JCFIV, the expression of linoleate 13 S-lipoxygenase 3-1, allene oxide synthase, and 12-oxophytodienoate reductase 3 in JA was upregulated. In pairwise JCFI vs JCFII and JCFIII vs JCFIV, the expression of adenylate isopentenyltransferase 5 (IPT5) in CK was upregulated. In pairwise JCFII vs JCFIII and JCFIII vs JCFIV, the expression of 3-ketoacyl-CoA thiolase 2 in JA was upregulated. Additionally, the expression of several genes gradually changed during ovule development. In pairwise JCFI vs JCFIII, CFI vs JCFIV or JCFII vs JCFIV, the expression of ent-kaurenoic acid oxidase 1 (KAO1) in GA and fatty acid beta-oxidation multifunctional protein AIM1 gene, 4-coumarate-CoA ligase-like 5, allene oxide cyclase 4 gene, linoleate 13 S-lipoxygenase 2-1 gene, and 12-oxophytodienoate reductase 11 gene in JA were upregulated, whereas cytosolic sulfotransferase 16, *ALDH3F1*, *YUCCA2* and *YUCCA4* in auxin, acyl-coenzyme A oxidase 2 gene in JA, phenylalanine ammonia-lyase gene in SA, and *CYP90D1* and *CYP90B1* in BR were downregulated (Table 2).

## Discussion

The embryo sac of *J*. *curcas* belongs to the polygonum type. A macrospore mother cell (MMC) only produces one functional macrospore that then develops into an embryo sac with 8 nuclei after three consecutive mitoses. Of these 8 nuclei, two move to the center of the embryo sac and form a polar, and the remaining six form an egg, two synergids, and three antipodal cells, respectively (Figs 1 and 2). A transcriptome covering the entire development process of sporogenesis and gametogenesis in both male and female flowers was constructed using Illumina sequencing.

Overall, plant hormone signal transduction declined during ovule development but showed a trend toward enhancement during the maturation of ES. Protein biosynthesis and processing were enhanced during the formation of MES. Sugar metabolism seemed to be enhanced during the development process of the ovule. A strong relationship exists between flower development and carbohydrates^12–15^. In particular, the concentration of starch in the ovule at specific steps of development is closely correlated with fertility^16^.

### Transcription factors participating in ovule development

Five TF families, such as bHLH, WRKY, MYB, *NAC* and ERF, would extensively contribute to ovule development, as suggested by the upregulation of 17 *bHLH*, 11 *WRKY*, 12 *ERF*, 10 *NAC* and 10 *MYB* during ovule development. The members of these five TF families are thought to be involved in ovule development. bHLHs play important roles in carpel, stigma and anther development and phytochrome signaling^17–19^. MYBs can regulate the development of anther, petal, and embryogenesis^20–22^. R2R3-MYBs are the largest group of plant MYB factors, and several R2R3-MYBs have been documented to function in ovule development. FOURLIPS and *MYB88* are thought to regulate megasporogenesis^23^, and *MYB98 is* specifically expressed in synergid cells^24^. For *WRKY*, *WRKY71* can accelerate flowering via the direct activation of *FT* and *LEAFY*^25^, and *WRKY34* and *WRKY2* are required for male gametogenesis in *Arabidopsis*^26^. Several members of the *AP2/ERF* family are thought to be involved in microspore, somatic and zygotic embryogenesis^27^. Members of the NAC family, such as CUC genes, contribute to ovule primordial development^28,29^.

### MADS-BOX, *SUP* and *SLK3* genes participate in ovule development

In the present study, five MADS-BOX genes *(AG-1*, *AG-2*, *AGL1*, AGL11 and *AGL14*), *SUP* and *SLK3* are expected to function in the development of the ovule in *J*. *curcas*. *AG* has been reported to determine stamen, carpel and ovule identity^30–32^. In this study, *AG-1* functioned during the stage of occurrence of MMC and *AG-2* throughout the entire development of the ovule. *SHP1* (*AGL1*) and *STK* (*AGL11*) are known as ovule identity genes that control ovule cell fate and regulate sporophyte and gametophyte development^33–35^. *SHP1* (*AGL1*) is also reported to function in regulating cell divisions in the ovule and in promoting stigma, style and medial tissue development^36,37^. Our results show that *AGL1* and *AGL11* play roles during the stage of MMC, controlling ovule cell fate, and regulating sporophyte development and cell divisions in the ovule. However, *AGL11* is also predicted to function during the formation of MES and regulate gametophyte development. AGL14 can control auxin transport via PIN transcriptional regulation^38^, and the PIN protein-dependent auxin gradient is responsible for pattern formation during early embryogenesis, organogenesis and ovule development^39^. Our results suggest that *AGL14* functions during the occurrence of MMC and regulates the early development of the ovule. *SUP* is known to function in the direct regulation of ovule development by dictating the growth of the adaxial outer-ovule integument by downregulating cell division^40–42^. In *J*. *curcas*, *SUP* may function during the occurrence of MMC when outer-ovule integument is not generated, and a new role of *SUP* in ovule development is predicted. Members of the *YABBY* family are active in determining abaxial-adaxial identity in *Arabidopsis* lateral organs^43^ and involved in amorphous or arrested growth of the integument^44^. However, *YABBY 5*, a member of the *YABBY* family, functions during the stages after the occurrence of MMC. The *SLK* gene is required for the proper development of vital female reproductive tissues derived from the carpel margin by maintaining meristematic potential in both the carpel margin meristem and the shoot apical meristem^45^. In the present study, *SLK3*, a member of the *SLK* gene, is predicted to function during the maturation of ES (a late stage of ovule development). Although these genes are predicted to function in certain developmental stages of the ovule, the details of their roles in ovule development are not clear and warrant further exploration. *CRC* has been thought to function in carpel morphogenesis and in the development of ovules^33,46^. Similarly, *CRC* was upregulated with the development of ovules in *J*. *curcas* and may function in the entire process of ovule development^47^.

### GA plays important roles at the stage from MMC in ovule development

GA is known to play various roles in plant reproductive development, and different species respond differently to GA^48^. The GA signaling pathway is involved in the development of stamens and pollen in *Arabidopsis* and tomato^49–51^. GA is a negative regulator in the development of stamen in maize^52,53^ but a positive regulator in *Arabidopsis* and rice^54^. GA is thought to play essential and complicated roles in floral organ development in *J*. *curcas*. In *J*. *curcas*, GA treatment increased both female and male flowers in the studies of Pan *et al*.^10^ and Gayakvad *et al*.^55^ promoted the development of stamens to produce bisexual flowers in research of Pi *et al*.^56^ and increased the number of female flowers in the research of Makwana *et al*.^57^. In gynoecious plants, GA treatment represses pistil development in female flowers to produce neutral flowers but does not resume the development of stamens^58^. In the present study, GA was predicted to promote the development of macrospores and embryo sacs in monoecious female flowers. The GA concentration in flower buds was increased at the stages from the occurrence of MMC to the maturation of ES, which was due to the upregulation of KO and KAO1. KO and KAO1 catalyzed the reaction to convert *ent* kaurene to GA_12_ during GA biosynthesis^59^. Furthermore, the upregulation of several GA-responsive genes (such as *GASL7 GASA3 GID1b and GAI*) during the stages from the formation of MMC to the maturation of SE may be the result of the increase in GA. In cotton, *GASL7* is predominantly expressed in cotyledons, and *GASA3* is expressed in fiber^60^. In *Arabidopsis thaliana*, *GASA3* accumulates in root, meristems, and flower seeds^61^. In *Arabidopsis thaliana*, 3 GA hormone receptors, such as *GID1a*, *GID1b*, and *GID1c*, have partially specialized functions in both proteolytic and nonproteolytic GA signaling. GID1b plays a stronger role in nonproteolytic GA signaling^62^. As a member of DELLA, GAI is a negative regulator of GA signaling^63^.

### JA plays important roles in the stage of maturation of EC

JA is thought to play critical roles in regulating reproductive development in plants, such as sex determination and stamen development in maize, and the development of inflorescences and flowers in rice and *Arabidopsis*^64^. In *J*. *curcas*, JA biosynthesis is lower in gynoecious than in monoecious florescence, indicating that the decrease of JA biosynthesis would likely contribute to the abortion of male flowers in gynoecious plants^60^. However, our results suggest that JA contributes to the maturation of EC in females; JA levels are increased during EC maturation, which is the result of the enhancement of JA biosynthesis, suggesting the upregulation of four JA biosynthesis genes, such as 3-ketoacyl-CoA thiolase 2, linoleate 13S-lipoxygenase 3-1, allene oxide synthase, and 12-oxophytodienoate reductase 3^59^. On the other hand, several JA-responsive genes, such as *MYC2*, *JAZ*, *TIFY 6B* and *TIFY 10B*, were upregulated at the maturation of EC, supporting our results. TIFY 10B and TIFY 6B belong to the JAZ subfamilies of the TIFY family and could be induced by JA^65,66^. In *Arabidopsis*, JAZ1/TIFY10b can work as a transcriptional repressor in the models of JA signaling^67,68^. Activation of JA response genes is mediated in part by *MYC2*^69–71^; in the absence of JA, MYC2’s function as a transcriptional activator is repressed by members of the JAZ family proteins, and elevated levels of JA induce the release of MYC2 from this repression by increasing the rate of degradation of JAZs^70,72^. Furthermore, MYC2 is a “master switch” in the crosstalk between JA and the other hormone signaling pathways^73^.

### Auxin may regulate the occurrence of MMC in ovule development

Auxin is thought to regulate ovule development^74,75^, possibly by determining the cell identity of the female gametophyte cell^39^, and the gradient of auxin is responsible for pattern formation during ovule development^39,76^. Similarly, our results also suggest that auxin contributes to ovule development. The auxin biosynthesis in flower buds is expected to be enhanced during the occurrence of MMC, as suggested by the upregulation of *YUCCA8*, *ALDH 3H1* and *ALDH 2B4-1*. In *Arabidopsis thaliana*, the *YUCCA* gene encodes a flavin monooxygenase-like enzyme that appears to oxidize tryptamine to N-hydroxytryptamine^77,78^. The ALDH family is thought to function in IAA biosynthesis by catalyzing the NAD-dependent formation of IAA from indole-3-acetaldehyde^79^. The expression levels of *SAURs* (22D, *10A5* and two *15A*), two *GH3s* (*GH3*.*17* and *GH3*.*1*), two *AUX/IAA* (*IAA1*, *IAA4*), *AFR3*, *ARG7-1*, *AIT12*, *ATLP2* and *5NG4* were upregulated during the development of the ovule, which would be associated with the elevated level of JA. Also, *10A5* and *15A* are members of *SAURs* and could be rapidly induced by auxin treatment within 2.5 min^80,81^. The *AUX/IAA* family is short-lived transcriptional factors and repressors of early auxin response genes at low auxin concentrations, and increased auxin could reduce the level of *AUX/IAA* proteins by accelerating their degradation. Different *GH3s* respond differentially to auxin treatment. In *Arabidopsis*, under IAA treatment, *GH3*.*1* showed strong transcriptional induction, while *GH3*.*5* and *GH3*.*6* showed weaker responses, and *GH3*.*17* showed little or no induction^82,83^. *AFR3* is a member of group II *AFRs* and is thought to be involved in floral meristem, gynoecium, stamen and perianth patterning in *Arabidopsis*^84^. Exogenous auxin treatment upregulates *ARF3*^85^.

### Cytokinin may regulate the occurrence of MMC and the maturation of ES in ovule development

In *Arabidopsis thaliana*, cytokinin is reported to regulate ovule formation and ovule number^86–91^ and to specify the functional megaspore in the female gametophyte^92^. In *J*. *curcas*, the application of CK increased the number of female flower^10,55^. Similarly, our results suggest that CK contributes to female development. CK biosynthesis in female flower buds would be enhanced at stages of MMC occurrence and ES maturation, as suggested by the upregulation of IPT and the downregulation of cytokinin dehydrogenase at these two stages. IPT catalyzes the initial step in the *de novo* biosynthesis of cytokinin in higher plants, whereas cytokinin dehydrogenase catalyzes the degradation of cytokinin^59^. In response to the increase in CK, several CK response genes, such as *HP4-2*, *HP5*, *HP1-2*, and five A-type *ARRs* (*ARR9-2*, *ARR5*, *ARR9-1* and two *ARR3*) were upregulated. The cytokine signaling cascade typically consists of three functional modules: HK, HP, and response regulator (ARR)^93^. HP proteins serve as phosphorelay carriers between cytokinin receptors and downstream nuclear responses, activating B-type *ARR* proteins, which in turn, activate A-type *ARRs*^94^. Additionally, A-type *ARRs* are reported to participate in ovule development. Megagametophyte defects could be observed in the *Arabidopsis* mutants arr7 and arr15^95^. In ovules lacking a functional embryo sac, *ARR7* and other A-type *ARRs* (*ARR4*, *ARR5* and *ARR6*) are overexpressed^96^. Most of the A-type *ARRs* could be rapidly and specifically induced by exogenous cytokinin^97,98^. *RRA3* and *RRA5* were robustly induced by cytokinin in *Arabidopsis* and *J*. *curcas*^2,10,99,100^.

### Brassinosteroids may function in the stages from meiosis of MMC to ES maturation

Brassinosteroids (BRs) suppress the development of the female flower in cucumber^101^, whereas they positively regulate the ovule number in *Arabidopsis* by regulating related gene expression by BZR1^102^. BRs influence ovule development by regulating the transcription of genes, such as *HUELLENLOS (HLL)* and *AINTEGUMENTA (ANT)*, which are redundant in the control of the ovule primordial growth^103^. *ANT* is a direct target of BRZ1, while *HLL* is regulated in an indirect way^102^. However, in this research, BR biosynthesis in flower buds is expected to decline during ovule development, as suggested by the downregulation of *CYP90D1* and *CYP90B1*. In *Arabidopsis*, *CYP90D1* plays an important role in the early C-22 oxidation of BR biosynthesis, and *CYP90B1* catalyzes multiple 22 *a*-hydroxylation steps in BR biosynthesis^104,105^. In contrast to the decline of BR biosynthesis, BR signaling-responsive DEGs were all upregulated during ovule development, such as *XTH22*, *BZR1*, *BAK1* and *BES1/BZR1*, indicating that cross-talk might exist between BRs and other phytohormone signaling pathways. Several reports proposed that BRs and GAs could interact at the signaling leve^106–108^. The DELLA protein GAI is thought to inactivate BZR1 by inhibiting the ability of BZR1 to bind to target promoters and negatively regulate BR signaling^106–108^. Additionally, auxin can also crosstalk with BRs through G-protein signaling^109^. XTHs, which can be upregulated by BRs, are thought to modify the length of xyloglucans, enabling the cell wall to expand^110,111^. BAK1 is a coreceptor of BRI1, and the binding of BRs to BRI1 relieves the repression of BKI and causes the association of BRI1 with BAK1, as well as a series of phosphorylation events^112,113^. BZR1 and BES1 are two major transcription factors regulated by BIN2 and mediate BR-regulated gene expression^77,114,115^.

## Conclusion

To the best of our knowledge, this report is the first to perform a transcriptome analysis of ovule development in *J*. *curcas*. A set of ovule development-related genes has been identified in female flower buds through expression profiling analysis, which provided comprehensive information on the ovule development of *J*. *curcas*, revealing that five TF families (*bHLH*, *WRKY*, *MYB*, *NAC* and *ERF*), five hormones signaling (auxin, GA, CK, BR and JA), five MADS-box genes *(AG-1*, *AG-2*, *AGL1*, *AGL11* and *AGL14*), *SUP* and *SLK3* participate in ovule development. GA and JA are involved in ovule development, as confirmed by their accumulation in flower buds during ovule development. Additionally, *CRC* and *SUP* are demonstrated to be involved in ovule development by qRT-PCR analysis. Transcriptome analysis is only an initial step in the exploration of the molecular mechanism of ovule development in *J*. *curcas*, but the ovule development-related genes identified in this study will establish a foundation for investigations into the molecular mechanisms of ovule development in *J*. *curcas*, and further analyses of these genes are needed to elucidate their roles in ovule development in *J*. *curcas*.

## Materials and Methods

### Flower bud samples collected

The flower bud samples of different development stages were collected from thirty *J*. *curcas* trees in May 2015 in Zhenfeng, southwestern China (36°14′50.2″N, 87°51′47.8″E). Each flower bud sample was separated into two portions. The portion used for the anatomic structure analysis of the ovary was fixed immediately in formaldehyde-acetic acid-50% alcohol mixtures (4: 6: 90, v/v), and the other portion used for transcriptome analysis was dipped immediately in RNAlocker (Tiandz, Inc., Beijing China) on ice. The flower bud samples used for the phytohormone quantification were stored in liquid nitrogen.

### Analysis of paraffin sections of ovaries

The flower bud samples used for the anatomic structure analysis of ovaries were fixed in formaldehyde-acetic acid-50% alcohol mixtures (4: 6: 90, v/v) for 24–48 h and then stored in 70% ethanol. The ovaries cut from fixed flower samples were dehydrated by gradient alcohol and then embedded in paraffin. Serial sections (8~10 μm thick) were cut using a Leica RM 2016 rotary microtome and stained in Safranin-Fast Green. The stained paraffin sections were analyzed under an Olympus – CX41 light microscope. The anatomic structure analysis of anther development will be discussed in detail in further research.

### Grouping on the flower bud samples and total RNA extraction

The flower bud samples stored in RNAlocker were then divided into eight development phases according to the morphology of flower buds at different development phases using an anatomical lens as follows: JCMI, the formation of ten stamen primordia; JCMII, from the occurrence of sporogenous cells to the occurrence of microspore mother cell; JCMIII, from the meiosis of microspore mother cell to the formation of meiotic tetrads; JCMIV, from free microspore stage to the maturation of pollen grain; JCFI, the growth of three carpels; JCFII, from the occurrence of sporogenous cells (SC) to the occurrence of macrospore mother cell (MMC); JCFIII, from the meiosis of MMC to the formation of mononuclear embryo sac (MES); JCFIV, from the formation of MES to the maturation of embryo sac (ES)(Figs 1 and 2).

For total RNA extraction, the flower buds from those eight development stages (each phase has three replicates) were ground in liquid nitrogen, and total RNA was isolated using the E.A.N. A Plant RNA Kit (Omega, USA) according to the manufacturer’s manual. The obtained RNA samples were then used for library construction.

### Library construction and Illumina sequencing

The RNA of flower buds from those eight flower development stages was applied for library construction. A total amount of 3 μg RNA per sample was used for the RNA sample preparations. RNA purity was checked using a NanoPhotometer® spectrophotometer (IMPLEN, CA, USA). RNA concentration was measured using a Qubit® RNA Assay Kit in Qubit® 2.0 Flourometer (Life Technologies, CA, USA). RNA integrity was assessed using the RNA Nano 6000 Assay Kit of the Agilent Bioanalyzer 2100 system (Agilent Technologies, CA, USA). Sequencing libraries were generated using the NEBNext® Ultra™ RNA Library Prep Kit for Illumina^®^ (NEB, USA) following the manufacturer’s recommendations, and index codes were added to attribute sequences to each sample. The quality of the library was assessed on the Agilent Bioanalyzer 2100 system and ABI Step OnePlus Real-Time PCR System in Novogene (Beijing, China). The clustering of the index-coded samples was performed on a cBot Cluster Generation System using the TruSeq PE Cluster Kit v3-cBot-HS (Illumina) according to the manufacturer’s instructions. After cluster generation, the library preparations were sequenced on an Illumina Hiseq platform, and paired-end reads were generated (Novogene Bioinformatics Technology, Beijing, China).

### *De Novo* Transcriptome Assembly and Abundance Estimation

The raw reads produced from sequencing machines were cleaned by removing reads with adaptors or unknown nucleotides larger than 10% and low quality with the percentage of low-quality bases (base quality ≤10) more than 50%. The left files (read1 files) from all libraries/samples were pooled into one large left. fq file, and right files (read2 files) into one big right. fq file. Transcriptome assembly was accomplished based on the left. fq and right. fq using Trinity^116^ with min-kmer-cov set to 2 by default and all other parameters set default. Transcriptome *de novo* assembly was carried out with the short read assembly program Trinity. The resulting sequences of trinity were called transcripts. When a gene has several transcripts, the longest one was selected as a unigene of the gene. Multiple samples from the same species are sequenced, and unigenes from each sample’s assembly can be taken into a further process of sequence splicing and redundancy removal with sequence clustering software to acquire nonredundant unigenes as long as possible.

### Unigene annotation and Protein - Coding Region Prediction

Gene function was annotated based on the following databases: NT (E-value ≤ 1.0E^−5^), NR, Swiss-Prot (E-value ≤ 1.0E^−5^), Pfam (Protein family) (E-value ≤ 0.01), KOG/COG (Clusters of Orthologous Groups of proteins) (E-value ≤ 1.0E^−3^), KO (KEGG Ortholog database) (E-value ≤ 1.0E^−10^) and GO (Gene Ontology) (E-value ≤ 1.0E^−6^).

For protein coding region sequence (CDS) prediction, unigenes were first aligned by blastx (E-value < 1.0E^−5^) to protein databases in the priority order of NR, Swiss-Prot. Proteins with the highest ranks in blast results were taken to decide the CDS of unigenes, and then both the nucleotide sequences (5′-3′) and amino sequences of the unigene CDS were acquired by translating the CDS into amino sequences with the standard codon table. Unigenes that cannot be aligned to any database were scanned by ESTScan (3.0.3) to obtain nucleotide sequences (5′-3′) and amino sequences of the CDS.

### Different expression genes (DGEs) determination

Clean read data of each sample were mapped back onto the assembled transcriptome. The resulting read count for each gene was obtained and then applied for gene expression level estimation in each sample using RSEM^117^. Differential expression analysis between two groups was performed using the DESeq R package (1.10.1). The resulting P values were adjusted using Benjamini and Hochberg’s approach for controlling the false discovery rate. Genes with an adjusted *P*-value < 0.05 were classified as differentially expressed genes (DEGs). The obtained DEGs were then subjected to KEGG enrichment analysis, and KOBAS software was used to test the statistical enrichment of differential expression genes in KEGG pathways^118^.

### Expression profile of genes involved in ovule development

Total RNA samples from flower buds at four different development phases (JCFI, JCFII, JCFIII and JCFIV) (Fig. 2) were used for expression pattern analysis of 16 selected DEGs (Table S5). The total RNA (1 µg) of each sample was used for first-strand cDNA synthesis using AMV RNA PCR Kit 3.0 (Takara). qPCR was performed on an ABI StepOnePlus Real-Time PCR System (Applied Biosystems, Inc. USA) using 2 × SYBR green PCR mix (QIAGEN, Shanghai, China). The β-tubulin gene was chosen as the endogenous reference gene for the qPCR analysis. The primers used for qPCR analysis are listed in Supplementary Table S1. Each sample has three biological replicates. The average threshold cycle (Ct) was calculated, and the relative expression level of each gene was then calculated according to the 2^−ΔΔCt^ method. A one-way ANOVA was performed on the gene expression level of the samples at different development stages using the software Statistical Package for the Social Science (SPSS) version 11.5 (SPSS Inc., Chicago, IL, USA). The individual treatment means were compared using the LSD (least significance difference) test.

### Quantification of phytohormone

The female flower bud samples at four development phases, JCFI, JCFII, JCFIII and JCFIV, were used for quantification of phytohormones. A 0.1-g sample frozen in liquid nitrogen was ground in liquid nitrogen and then dipped in 1 mL mixture composed of cold acetone: deionized water: hydrochloric acid (37%) = 2:1:0.002 (V:V:V). After incubation at 4 °C for 30 min, 1 mL dichloromethane was added. After incubation at 4 °C for 30 min, the sample was then centrifuged for 15 min at 13,000 g and 4 °C, and the resulting subnatant was collected for drying by liquid nitrogen. Phytohormones in the dried residue were extracted with 0.1 mL methanol. Quantification of phytohormones was performed as described previously using a liquid chromatography–mass chromatography system (Shiseido SP HPLC- Thermo TSQ Quantum Ultra MS/Ms) with an ODS column (SHISEIDO C18, 5 μm, 2.0 × 150 mm)^119,120^,^121^.

Availability of Supporting Data All RNA-Seq data for this project has been deposited in NCBI under the SRA accession SRP115141. Summary description of library Illumina sequencing, *De novo* transcriptome assembly Table S1 and annotation are listed in Supplementary Data Tables S2–S5, Figs S1–S7.

## Supplementary information

The revised version of supplementary information.pd
