# Supplementary material for: Comparative gene expression profile analysis of ovules provides insights into Jatropha curcas L. ovule development
Source: Sci Rep. 2019 Nov 4;9:15973. doi: 10.1038/s41598-019-52421-0 (PMC6828956; doi:10.1038/s41598-019-52421-0)
Supplement: Supplementary file 1 — The revised version of supplementary information.pd [file 41598_2019_52421_MOESM1_ESM.pdf]

Submitted to Scientific Reports

Comparative gene expression profile analysis of ovules provides insights into *Jatropha curcas* L. ovule development

Gang Xu[1,4*], Jian Huang[3], Shi-kang Lei[2], Xue-guang Sun[1], Xue Li[2]

1. Institute for Forest Resources and Environment of Guizhou, Guizhou University, Guiyang 550025, P.R. China

2. School of Life Science, Guizhou University, Guiyang, Guizhou, P.R. China

3. Key Laboratory of Green Pesticide and Agricultural Bioengineering, Ministry of Education, Center for Research and Development of Fine Chemicals of Guizhou University, Guiyang, Guizhou, P.R. China

4. Institute of Entomology, Guizhou University, Guiyang, Guizhou, P.R. China

*Corresponding author:

Gang Xu

E-mail: xg335300@aliyun.com

Supporting Information

Fig. S1 Length distribution of the transcripts and unigenes from the sample.

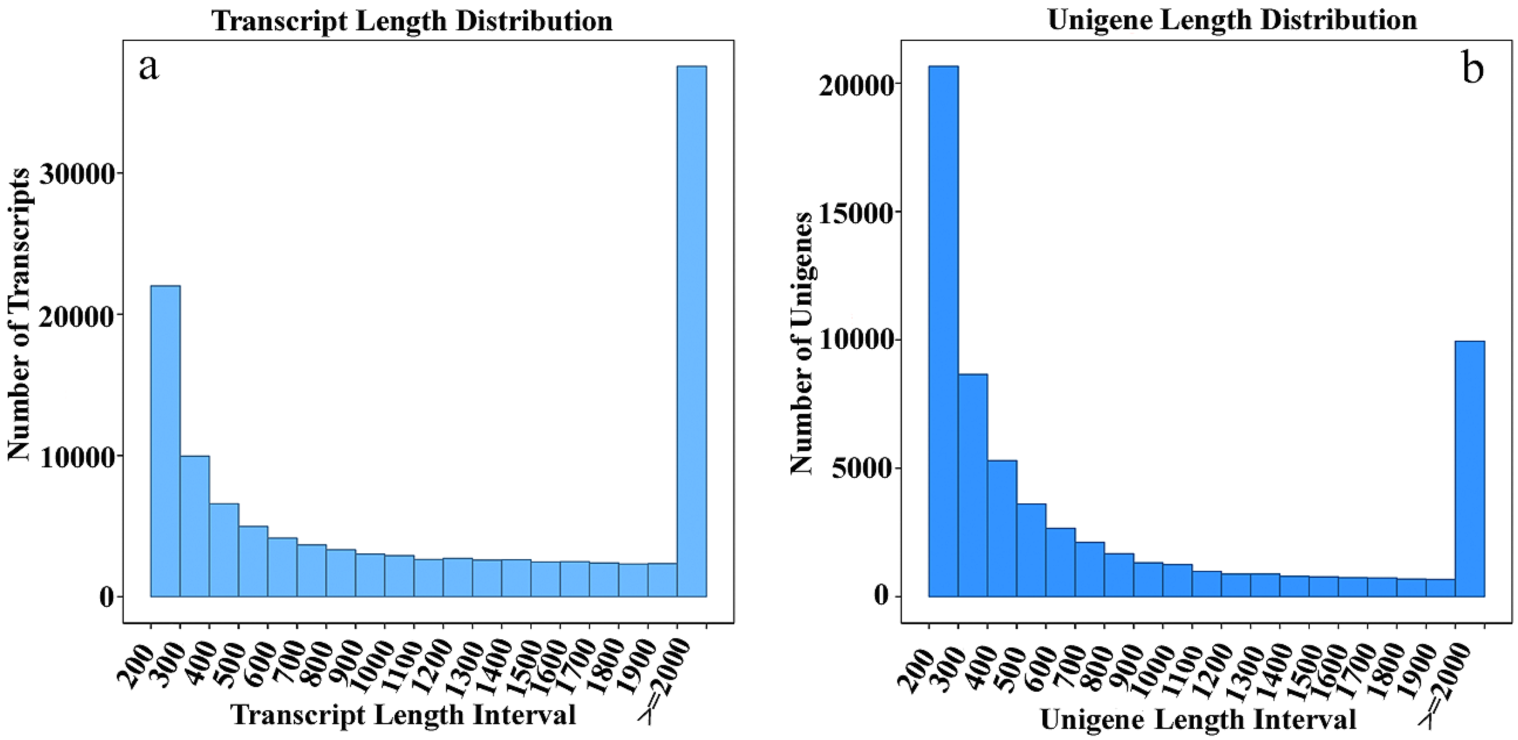

Fig. S2 Length distribution of CDS and peptides.

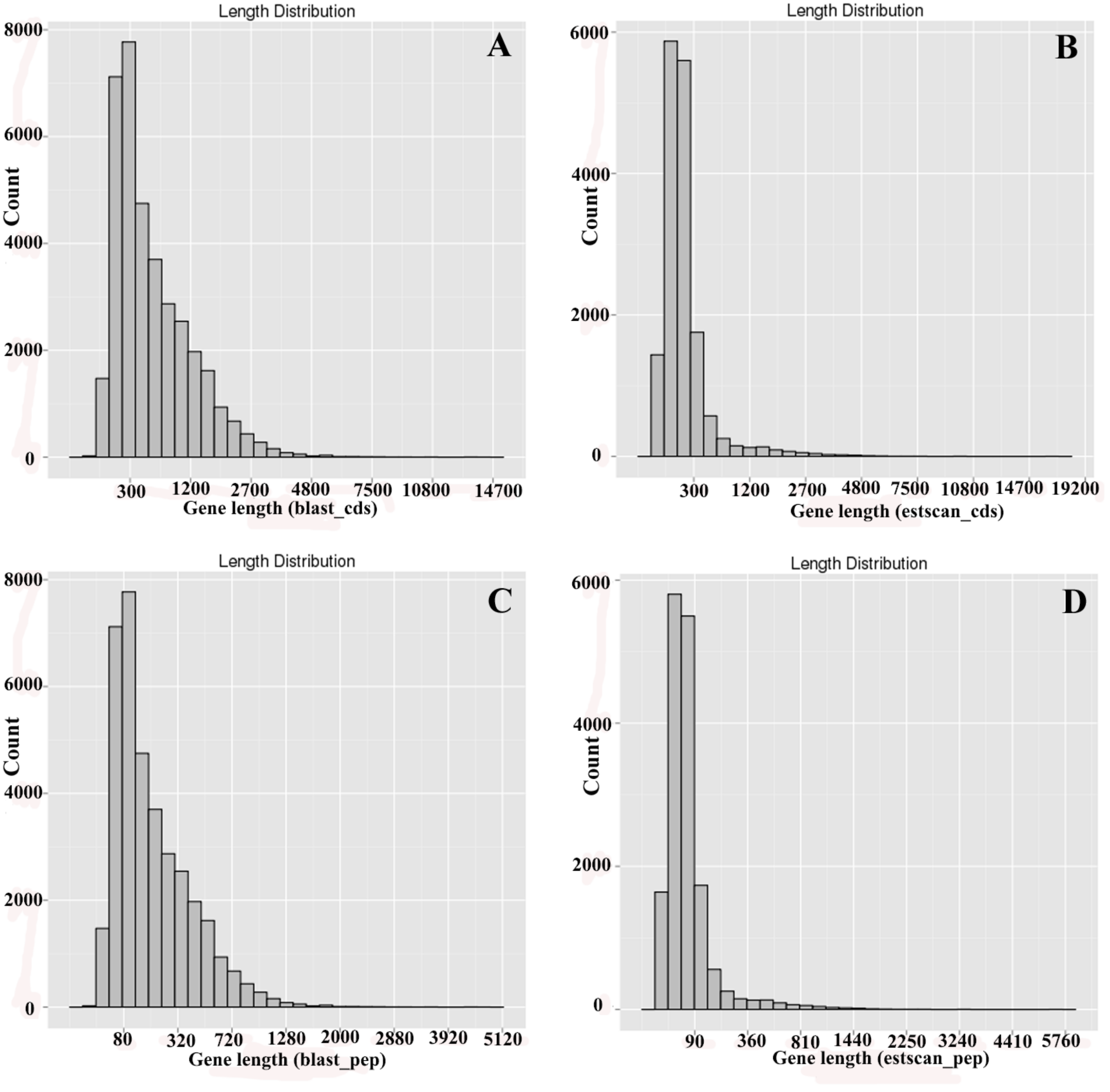

Fig. S3 Data of NR classification. (a) E-value distribution of BLAST hits for each unique sequence with a cut-off E-value of $1.0E^{-5}$. b) Similarity distribution of the top BLAST hits for each sequence. (c) The species distribution is shown as a percentage of the total homologous sequences, with an E-value of at least $1.0E^{-5}$.

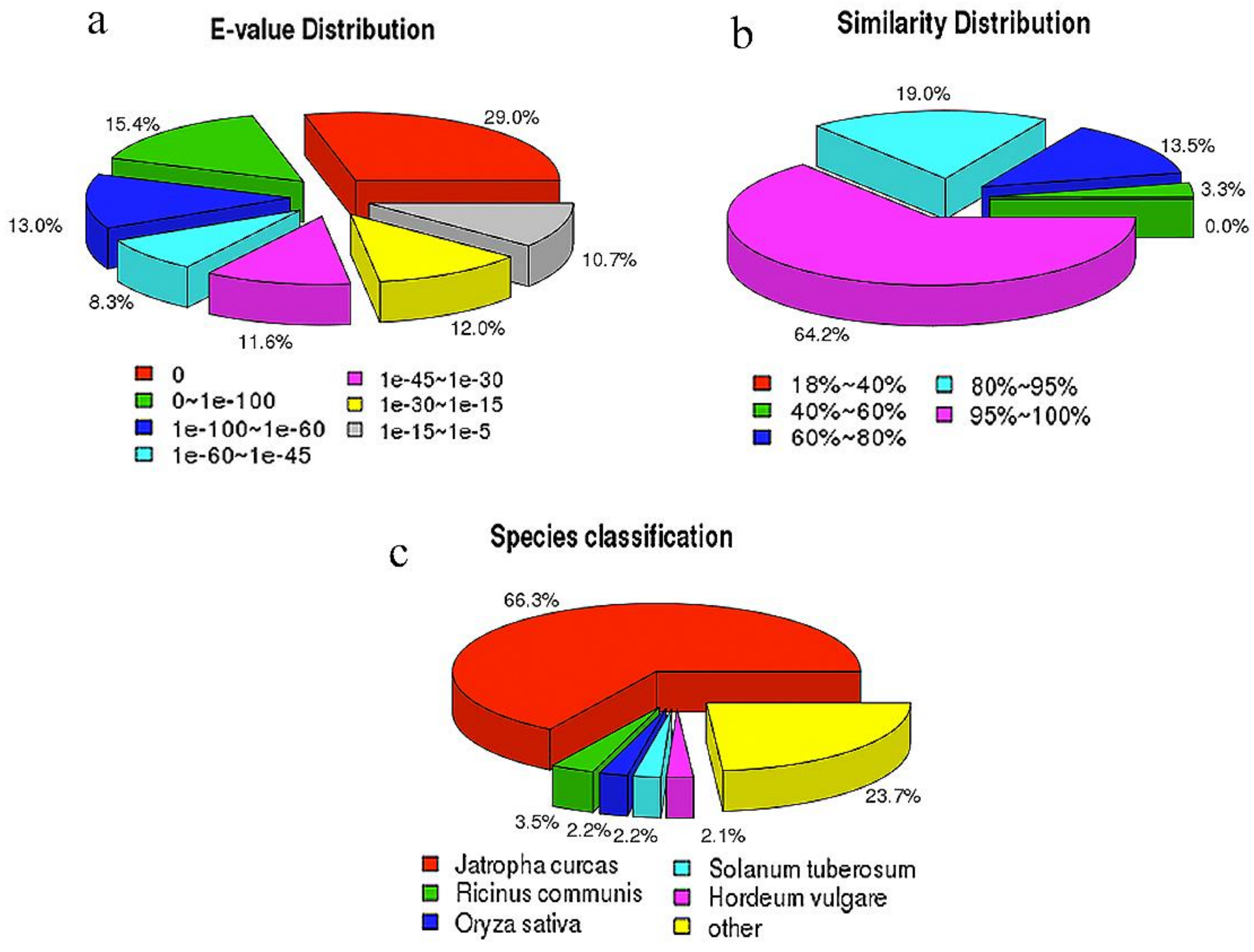

Fig.S4 KOG function classification of the Unigenes of transcriptome.

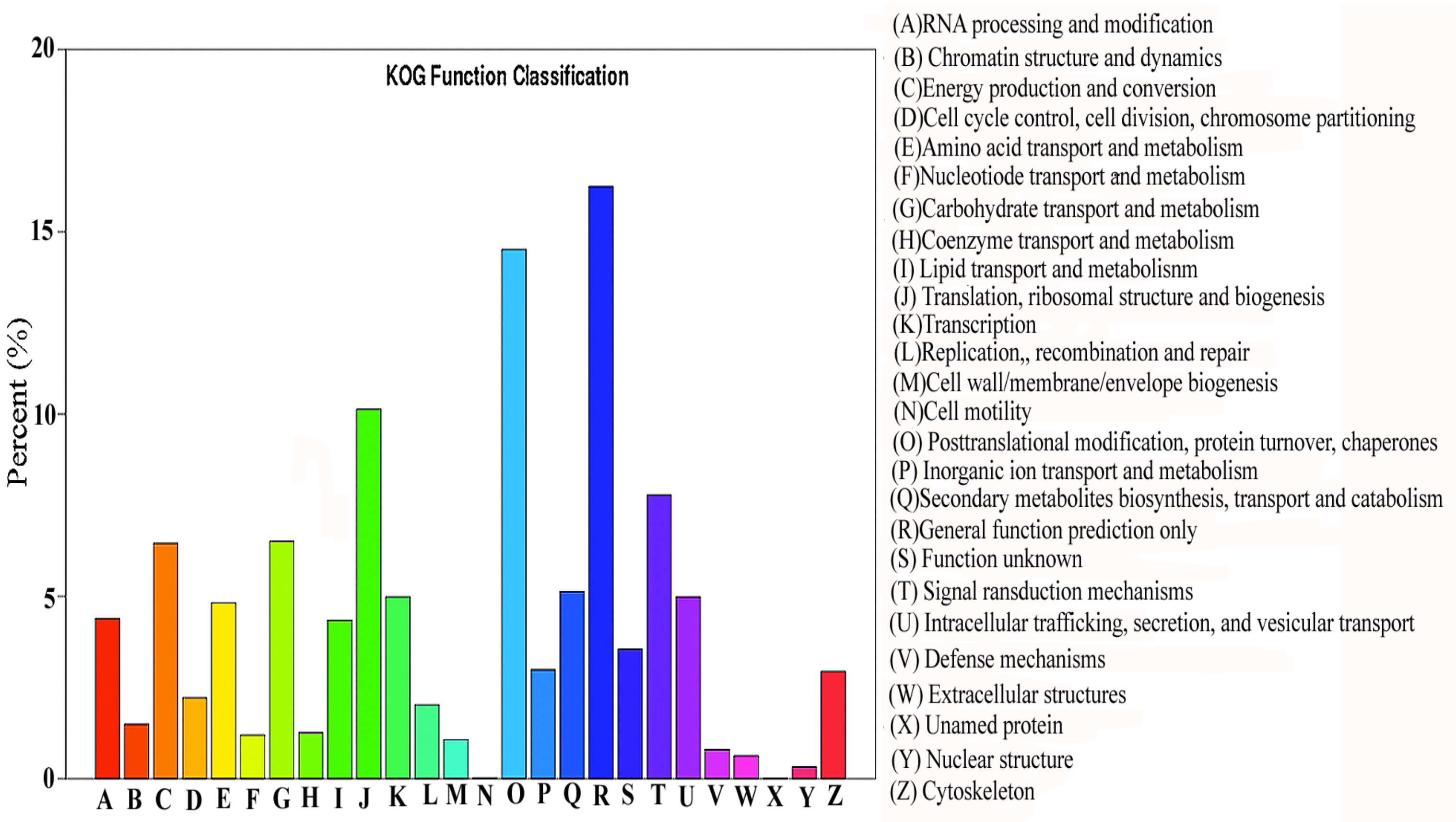

Fig.S5 GO function classification of the Unigenes of transcriptome.

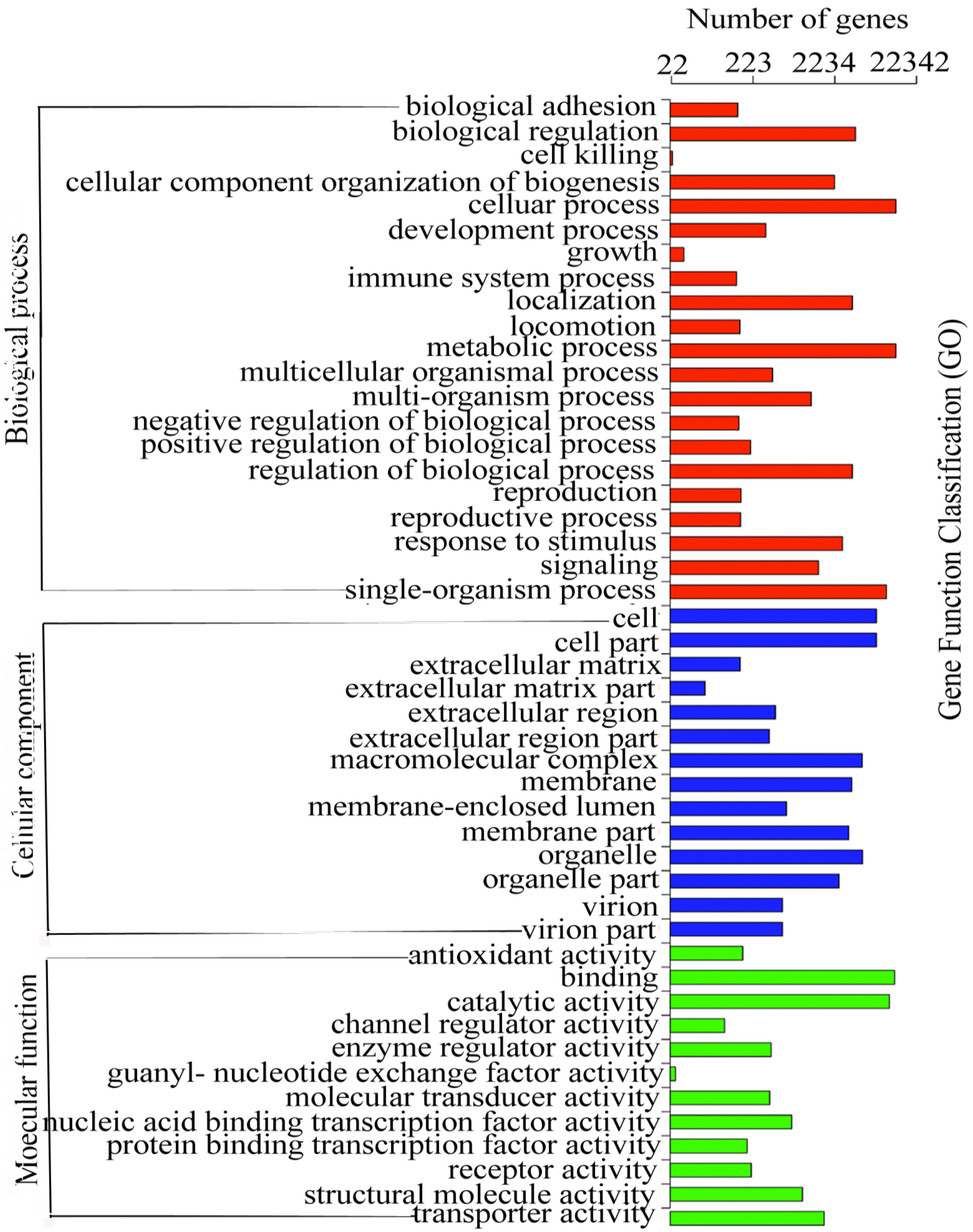

Fig.S6 KEGG pathways analysis of the Unigenes of transcriptome.

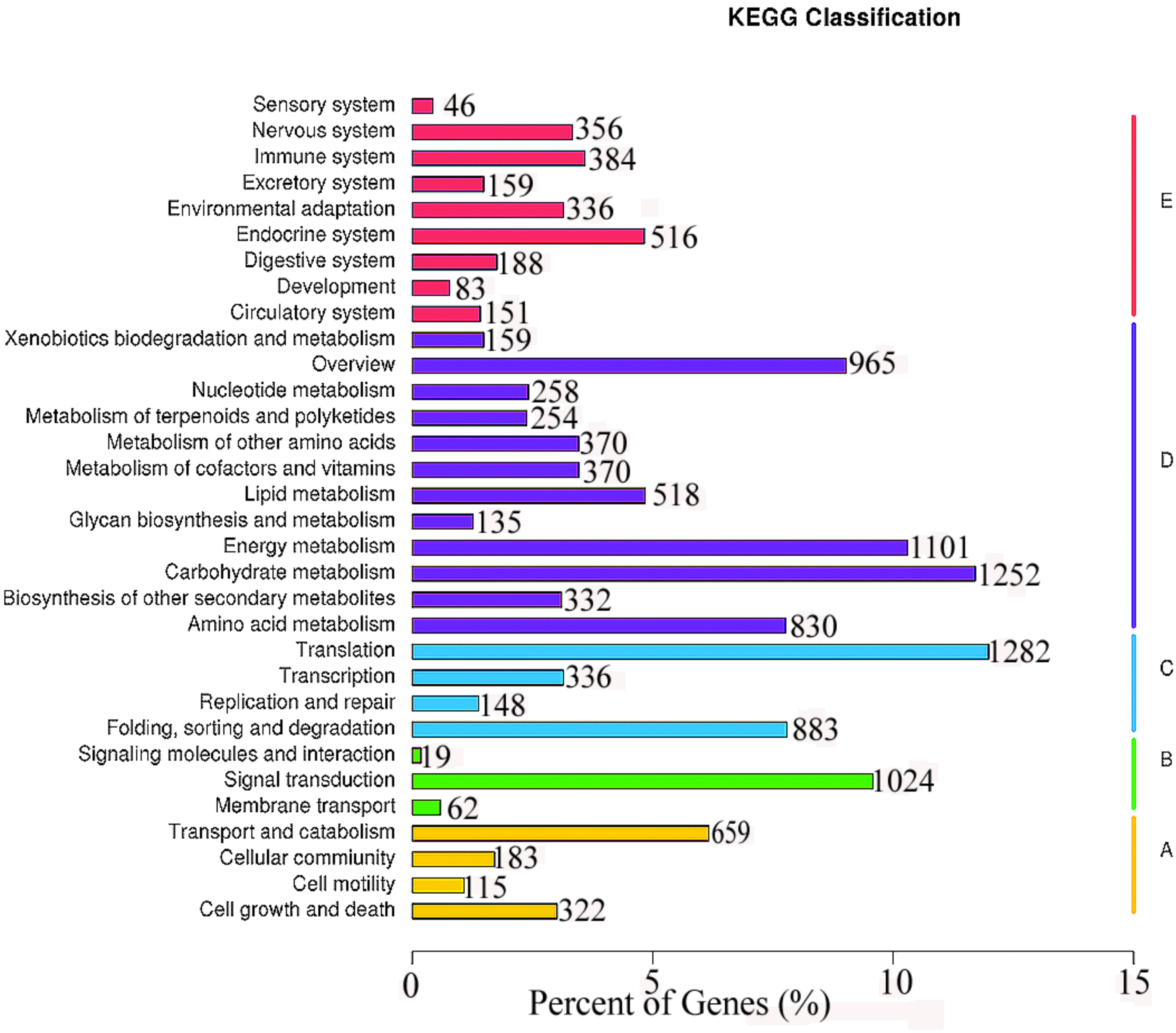

Fig. S7 Reads uniform distribution of the transcript with the distance from 5`end of the twelve samples.

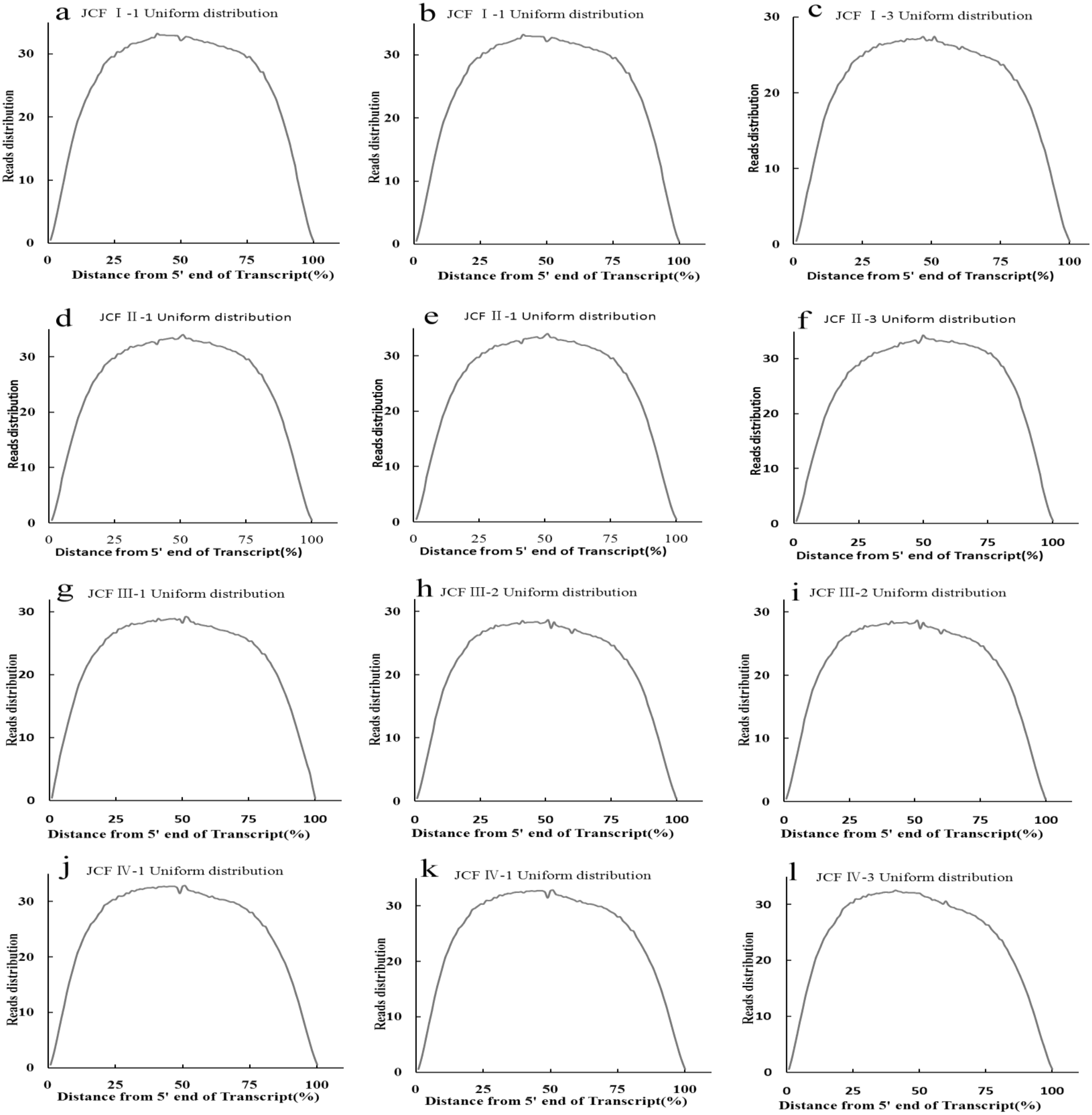

Table S1 Primers of the 15 selected unigenes and the reference gene (Tubulin beta) used for validation of expression trend.

	Gene ID	Primer (5'to3')
	c28570_g1-F	CAAAGGCCAAAGCCCAAAGT
1	c28570_g1-R	ATGCTGGCAATGTTTCGGAC
	c26065_g1-F	TAGCTTCACAACCGCAAGGA
2	c26065_g1-R	ACTTACCCTGTGGACGGAGG
	c27617_g8-F	TGGTCAATGGAGCACAAACC
3	c27617_g8-R	ATGCGTACCACAACTCCGAA
	c25603_g2-F	CACGATTGCGTCACCTGAAT
4	c25603_g2-R	TCGACATGGCTTCTGCACT
	c8047_g1-F	TCTGCAGTGATGGTGCGTCT
5	c8047_g1-R	GCCACCTTCATTCCTCCTCC
	c30007_g3-F	CTGCAGTGACCGCATTTCAC
6	c30007_g3-R	CTCTGCTATGTCCGCTGCAA
	c29827_g2-F	CGTGGAAAGATGCGAGGTGT
7	c29827_g2-R	TCTCGCTTTCATCGACTCGG
	c29219_g1-F	CCATGATGCAAACTGTCACG
8	c29219_g1-R	AATGGAGAAGCTCCCTTGGA
	c24288_g1-F	ACCGCGGCTAGAGAATACGA
9	c24288_g1-R	TCGCCAAGTCACTTTCTGCA
	c15640_g1-F	GCGGTACCTGCTGCTTTAGG
10	c15640_g1-R	TTGTTGGCGTGAGTCGTCAT
	c28499_g1-F	TCAATGGCGGATACTGGAGG
11	c28499_g1-R	AGCGTAGCCAAACTGCAAGG
	c11413_g1-F	GTCGGTCACATGAGAGGCAA
12	c11413_g1-R	ACAGCCCTACGATGTGTCCA
	c21569_g1-F	TTCGTTCCGATTTTCGCTCC
13	c21569_g1-R	ACACCACCTCCGGCATGAAT
	c25410_g1-F	GCGCTCTATCGCCTGTTCTT
14	c25410_g1-R	ACCGACTCGAACAAGGAAGG
	c21626_g1-F	CACTCATCTGTTGCCCGCTA
15	c21626_g1-R	TACTGACAACCGAACAGCCG
	c22538_g1-F	TCCGGTAAGGCTGTAACCCC
16	c22538_g1-R	GCGACGCTGATGTTGCTCTT
	Tubulin beta-F	GCCCCACTTACTTCTCGTGG
17	Tubulin beta-R	GGCAGTTAAGTAGCGGCCAT

Table S2 Summary of reads quality from each sample.

Sample	Raw Reads	Clean Reads	Clean Bases	Error(%)	Q20(%)	Q30(%)	GC Content(%)
JCMI_1	30123010	29170474 (96.8%)	3.65G	0.02	96.61	92.02	42.88
JCMI_2	36069556	34851626 (96.6%)	4.36G	0.02	96.37	91.55	42.87
JCMI_3	28003324	27140556 (96.9%)	3.39G	0.02	96.54	91.89	42.91
JCMII_1	29764100	28813978 (96.8%)	3.6G	0.02	95.71	90.33	42.21
JCMII_2	32703002	32044924 (98.0%)	4.01G	0.02	95.59	89.93	42.52
JCMII_3	26663678	25675618 (96.3%)	3.21G	0.02	95.71	90.29	42.38
JCMIII_1	31655964	30405248 (96.0%)	3.8G	0.02	95.94	90.81	42.24
JCMIII_2	27334974	26253172 (96.0%)	3.28G	0.02	95.87	90.65	42.14
JCMIII_3	30949432	28873140 (93.3%)	3.61G	0.02	96	90.83	42.43
JCMIV_1	31883006	28199808 (88.4%)	3.52G	0.02	95.63	90	43.29
JCMIV_2	32853878	30815506 (93.8%)	3.85G	0.02	95.63	90.02	43.6
JCMIV_3	34772570	31132474 (89.5%)	3.89G	0.02	95.6	90.03	43.39
JCFI_1	29580132	29183380 (98.7%)	3.65G	0.02	96.27	91.33	43.56
JCFI_2	28279310	27047964 (95.6%)	3.38G	0.02	96.25	91.29	43.15
JCFI_3	24828278	23804096 (95.9%)	2.98G	0.02	96.12	91.01	43.23
JCFII_1	29899422	28916134 (96.7%)	3.61G	0.02	95.93	90.64	43.06
JCFII_2	29540294	29081016 (98.4%)	3.64G	0.02	95.4	89.46	42.9
JCFII_3	30129444	29132620 (96.7%)	3.64G	0.02	96.1	91.02	42.89
JCFIII_1	25351052	23639788 (93.2%)	2.95G	0.02	95.97	90.77	42.89
JCFIII_2	26500748	25380100 (95.8%)	3.17G	0.02	96.11	90.98	43.17
JCFIII_3	29642364	27769024 (93.7%)	3.47G	0.02	96.29	91.37	43.21
JCFIV_1	29535236	28382156 (96.1%)	3.55G	0.02	95.99	90.78	42.74
JCFIV_2	29748550	28839698 (96.9%)	3.6G	0.02	95.81	90.41	42.78
JCFIV_3	29314160	28701200 (97.9%)	3.59G	0.02	95.78	90.29	43.00

Table S3 Distribution of transcript and unigene length.

Transcript length interval	Number of transcripts	Number of Unigenes	Statistics of transcript length distribution	Transcripts	Unigenes
200-500bp	38,593	34,647	min length	201	201
500-1kbp	19,094	11,346	mean length	1612	1000
1k-2kbp	25,425	8,390	median length	1091	448
>2kbp	37,546	9,942	max length	17391	17391
Total	120,658	64,325	N50	2787	2146
			N90	787	346
			total nucleotides	194,527,365	64,355,776

Table S4 Summary of unigene annotation.

Data base	Number of Unigenes	Percentage (%)
Annotated in NR	30736	47.78
Annotated in NT	25334	39.38
Annotated in KO	10696	16.62
Annotated in SwissProt	22570	35.08
Annotated in PFAM	21930	34.09
Annotated in GO	22342	34.73
Annotated in KOG	12332	19.17
Annotated in all Databases	5630	8.75
Annotated in at least one Database	36264	56.37
Total Unigenes	64325	100

Table S5 Summary of the clean reads mapped to transcription in each sample

Sample name	Total clean reads	Total mapped
JCFⅠ_1	29183380	24449388(83.78%)
JCFⅠ_2	27047964	23530440(87.00%)
JCFⅠ_3	23804096	20697442(86.95%)
JCFⅡ_1	28916134	24919562(86.18%)
JCFⅡ_2	29081016	25239518(86.79%)
JCFⅡ_3	29132620	25196404(86.49%)
JCFⅢ_1	23639788	20312148(85.92%)
JCFⅢ_2	25380100	22045930(86.86%)
JCFⅢ_3	27769024	24114666(86.84%)
JCFⅣ_1	28382156	24651498(86.86%)
JCFⅣ_2	28839698	24917762(86.40%)
JCFⅣ_3	28701200	24425054(85.10%)
